# Solvent Environment Influences Molecular Conformation and Electron Transport in Peptides

**DOI:** 10.1021/acs.jpclett.6c01257

**Published:** 2026-05-17

**Authors:** Rajarshi Samajdar, Hassan Nadeem, Neil Moghe, Diwakar Shukla, Charles M. Schroeder

**Affiliations:** † Department of Chemical and Biomolecular Engineering, 14589 University of Illinois Urbana-Champaign, Urbana, Illinois 61801, United States; ‡ Beckman Institute for Advanced Science and Technology, 14589 University of Illinois Urbana-Champaign, Urbana, Illinois 61801, United States; § Department of Bioengineering, 14589 University of Illinois Urbana-Champaign, Urbana, Illinois 61801, United States; ∥ Center for Biophysics and Quantitative Biology, 14589 University of Illinois Urbana-Champaign, Urbana, Illinois 61801, United States; Department of Materials Science and Engineering, 14589 University of Illinois Urbana-Champaign, Urbana, Illinois 61801, United States; # Department of Chemistry, 14589 University of Illinois Urbana-Champaign, Urbana, Illinois 61801, United States; □ Department of Chemical and Biological Engineering, Princeton University, 35 Ivy Lane, Princeton, New Jersey 08540, United States

## Abstract

Hierarchical structures play a key role in governing the electronic properties of peptides. Despite recent advances, establishing clear structure–property relationships that connect the solvent environment, molecular conformation, and electron transport at the single-molecule level remains challenging. Here, we use a combination of single-molecule experiments, molecular dynamics (MD) simulations, and machine learning (ML) analysis to understand how electron transport in peptides depends on solvent conditions for several different environments including water, 2,2,2-trifluoroethanol, acetonitrile, and glycerol. Our results reveal two distinct conductance populations for peptides in water or 2,2,2-trifluoroethanol: a high-conductance state associated with defined secondary structures (β turns or 3_10_ helices) and a low-conductance state corresponding to extended primary structures. Peptides show a diminished high-conductance state in acetonitrile, which is known to weakly stabilize secondary structures and denature peptides. Interestingly, the high-conductance state is diminished in glycerol for tetrapeptides but not for pentapeptides. Unsupervised ML analysis using silhouette clustering and Gaussian mixture modeling suggests that solvent-dependent conductance behavior is mediated by peptide conformation. Complementary MD simulations, time-lagged independent component analysis of intramolecular hydrogen-bonding (H-bonding) distances, and Pearson correlation coefficients further reveal how solvent-peptide interactions and secondary structures govern electron transport pathways. Overall, our results show that the solvent environment significantly influences electron transport in peptides mediated by secondary structure and H-bonding interactions.

## Graphical abstract

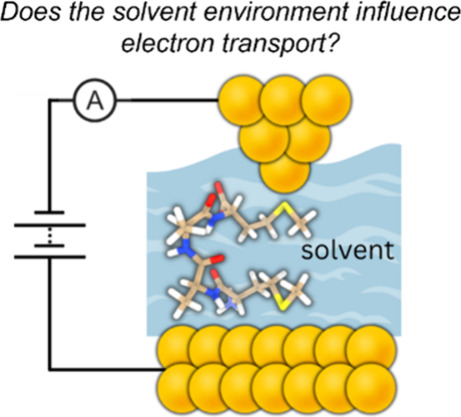

Understanding electron transport in biomolecules such as peptides is critical for advancing new technologies in bioelectronics,^1^ biomedical devices,^2^ and molecular sensors.^3^ Prior work has focused on a combination of experiments, theory, and simulations to characterize electron transfer reactions in complex biological systems, ranging from redox events in metalloproteins^4–6^ and redox-active cofactors^7,8^ to metal-reducing bacteria.^9^ During redox-mediated electron transfer events, intervening amino acid residues between redox centers are thought to provide a conductive matrix for electron transport.^10^ Recent work has shown that the electronic properties of peptides depend on the molecular conformation of peptide backbones, with a high-conductance state arising due to a defined secondary structure (β turn or 3_10_ helices) and a low conductance state occurring due to an extended primary structure.^11^ Despite recent progress, the role of the solvent environment on the molecular conformation and corresponding electronic properties of peptides is not yet fully understood. Although solvent effects on peptide conformation are known, how hydrogen-bonding capability and denaturing propensity influences single-molecule electron transport is not fully understood.

Understanding the mechanisms of electron transport in model peptides and proteins offers valuable insights into how the local solvent environment influences the electronic properties in more complex hierarchical protein assemblies. The dominant mechanism of electron transport in short peptides has been reported to be nonresonant coherent tunneling,^12–20^ where conductance decays exponentially with molecular length. In contrast, electron transport in larger biomolecules such as proteins can occur via hopping,^7,21^ where conductance decreases inversely with distance. It is known that variations in the local environment around the donor or acceptor centers affect the driving force for the electron transfer reaction and the reorganization energy, in accordance with Marcus theory.^22^ Additionally, changes in the local environment directly affect electronic coupling through biomolecules by influencing the molecular conformation and hydrogen bonding (H-bonding) interactions. However, key knowledge gaps remain in understanding how the solvent environment alters peptide conformation and H-bonding interactions to enhance or suppress electron transport for nanoscale electron tunneling.

In this work, we use a combination of single-molecule electronic measurements and computational modeling to characterize the role of solvent environments on peptide electron transport. The electron transport properties of peptide sequences MAAM and MAAAM were studied using the scanning tunneling microscope-break junction (STM-BJ) technique^23–25^ in various solvents including water, 2,2,2-trifluoroethanol (TFE), acetonitrile (ACN), and glycerol. Our results reveal a bimodal conductance distribution for peptides in water and TFE, with a high-conductance state arising due to a defined secondary structure (β turn or 3_10_ helices) and a low conductance state arising due to an extended primary structure. Interestingly, the high-conductance state is selectively diminished in acetonitrile, whereas in glycerol, it is diminished in tetrapeptides but not in pentapeptides. Unsupervised machine learning (ML) algorithms based on silhouette clustering and Gaussian mixture modeling (GMM) are used to show that peptides in various solvents undergo conformation-mediated electron transport. Molecular dynamics (MD) simulations are further used to understand H-bonding interactions of peptides in various solvents. Time-lagged independent component analysis (tICA) is used to characterize the peptide conformational landscape. In contrast to tetrapeptides, which primarily exhibit a 1 → 4 H-bond mediated electron transport pathway, pentapeptides exhibit multiple potential H-bond interactions along the backbones (1 → 4, 1 → 5, and 2 → 5 interactions). Pearson correlation coefficients (PCCs) are used to quantify the contribution of each potential H-bonding transport pathway to the slowest dynamic process identified using tICA, thereby revealing the dominant transport pathways for pentapeptides. Results from MD simulations, tICA analysis, and computed PCC values are used to rationalize results from single-molecule electron transport experiments. Overall, our work shows that solvent significantly influences electron transport in short peptides, providing new insight into understanding the role of solvent environment in peptides and proteins.

Tetrapeptide and pentapeptides (Supplementary Figures 1–2) featuring nonpolar aliphatic side groups (MAAM and MAAAM, respectively) were characterized in four different solvents (water, glycerol, 2,2,2-trifluoroethanol, and acetonitrile) to understand the role of solvent environment on electron transport (Figure 1). Water has a high dielectric constant^26^ and was used as a reference to compare results with prior reports on the molecular electronic properties of peptides (Figure 1a).^11^ Glycerol has an extensive capacity for H-bonding interactions^27^ and offers a different local environment than water^28^ (Figure 1b). 2,2,2-trifluoroethanol (TFE) is known to promote secondary structure formation^29–31^ in peptides and proteins (Figure 1c), which could stabilize molecular conformations conducive to electron transport. In contrast, acetonitrile (ACN) only weakly stabilizes secondary structures^32,33^ and is largely denaturing (Figure 1d),^34^ which could perturb hierarchical structures.

**Figure 1:**
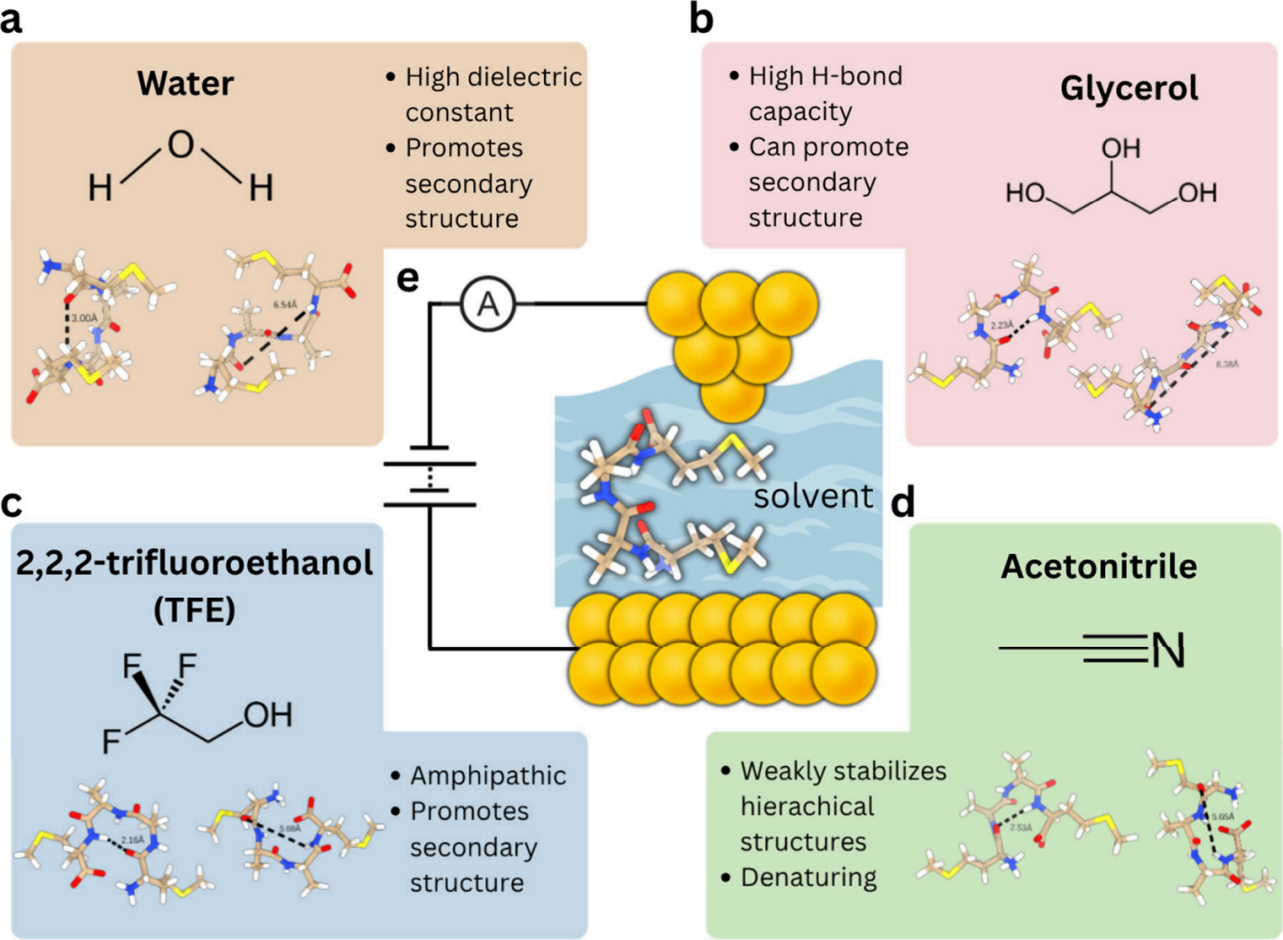
Investigating the role of solvent environments on molecular conformation and electron transport in peptides. (a) Water exhibits high dielectric constant, facilitating polar interactions and electrostatic screening. (b) Glycerol possesses high H-bonding capacity and promotes protein stability through preferential hydration. (c) 2,2,2-trifluoroethanol (TFE) acts as an amphipathic solvent that promotes secondary structure formation by weakening intramolecular hydrogen bonds. (d) Acetonitrile weakly stabilizes hierarchical protein structures and can induce protein denaturation. (e) Schematic representation the STM-BJ setup.

Circular dichroism (CD) was used to characterize the secondary structure of peptides in different solvents. CD spectral features for 3_10_ helices are qualitatively different than the spectral features observed for alpha helices, beta sheets, or random coils.^35^ CD spectra for MAAM and MAAAM in water are consistent with the formation of 3_10_ helices,^36,37^ as reported in prior work,^11^ characterized by distinct features around ∼200–210 nm and a shoulder or small peak around ∼220 nm (Supplementary Figure 3).^36,37^ The CD spectra of MAAM and MAAAM in TFE indicate the presence of H-bonding interactions but exhibit markedly different spectral line shapes and intensities compared to those observed in water (Supplementary Figure 4). The CD spectra of MAAM in ACN indicates opposite helicity compared to MAAM in water, whereas the CD spectra of MAAAM in ACN is significantly diminished, suggesting that ACN may inhibit intramolecular H-bonding interactions in peptides with nonpolar amino acids (Supplementary Figure 5). The acquisition of CD spectra in pure glycerol was not feasible due to the intrinsic absorbance of the solvent, and hence CD spectra were determined in glycerol–water mixtures with low glycerol content (<10%) (Supporting Information Section S1). Our results indicate that MAAM exhibits reduced intramolecular H-bonding in the presence of glycerol (Supplementary Figure 6), whereas MAAAM retains the ability to form H-bonds under the same conditions. Overall, results from CD spectra reveal clear differences in H-bonding interactions between MAAM and MAAAM arising in different solvent environments.

The N- and C-terminal residues of the peptides were selected as methionine, which contains a thiomethyl (-S-CH_3_) group that readily binds to gold,^38^ thereby providing electrical contacts to metal electrodes for scanning tunneling microscope-break junction (STM-BJ) experiments (Figure 1e). The STM-BJ setup consists of a gold tip electrode that is repeatedly moved into and out of contact with a gold substrate electrode in a solution (water, acetonitrile, 2,2,2-trifluoroethanol, and glycerol) containing molecules, resulting in the continual formation and breakage of single-molecule junctions. The STM-BJ instrument is automated, and experiments are repeated over an ensemble of >5000 molecules for each experiment. Single-molecule conductance data are then analyzed using one- and two-dimensional (1D and 2D) conductance histograms without data selection. In 1D conductance histograms, all recorded conductance values over the course of the measurement are compiled, and the peak of the histogram represents the most probable conductance value. 2D molecular conductance histograms show the distribution of conductance values together with junction separation distances, providing insights into the evolution of conductance as the junction is extended. The time scale of a single STM-BJ pulling trajectory is in the order of milliseconds,^39^ which allows for sampling a range of molecular conformations^11,24^ during a conductance measurement. Prior work^11^ demonstrated that electron transport in short peptides such as MAAM and MAAAM is conformation-dependent in water, with a high-conductance state arising from a defined secondary structure (β turns or 3_10_ helices) and a low-conductance state corresponding to extended peptide conformations (Supplementary Figure 7).

We began by performing STM-BJ experiments on the tetrapeptide MAAM in different solvents. 1D and 2D molecular conductance histograms (Figures 2a,b) indicate that the bimodal conductance histogram observed in water for MAAM is also observed in TFE, which is known to promote secondary structure formation in peptides and proteins.^29–31^ Interestingly, the high conductance state arising due to H-bonding interactions is significantly diminished in glycerol and acetonitrile (Figure 2a,c and Supplementary Figure 8). These results suggest that solvent-dependent stabilization or disruption of intramolecular H-bonds, and the denaturing capabilities of solvent, influence electron transport. We next performed STM-BJ experiments on the pentapeptide MAAAM in different solvents. Our results show a bimodal conductance behavior in TFE and water, which is greatly diminished in acetonitrile, similar to the experimental observations for the alanine-based tetrapeptide sequence MAAM (Supplementary Figures 9a–c). Although the dielectric constant describes bulk electrostatic screening, it does not fully capture solvent-peptide interactions at the molecular scale. Empirical solvent parameters such as E_T_(30),^40,41^ which reflect local solvation and H-bonding ability, provide a relevant framework for interpreting peptide conformational behavior (Supplementary Table 1). Water and TFE exhibit similar E_T_(30) values despite large differences in dielectric constants, and both stabilize H-bonded peptide conformations. In contrast, acetonitrile has a lower E_T_(30) value and weak H-bond donor capability, consistent with its reduced ability to stabilize folded conformations. Interestingly, the high conductance state for MAAAM in glycerol appears to be present (Supplementary Figure 9d), whereas it is significantly diminished in MAAM (Figure 2c), consistent with CD results showing negligible H-bonding for MAAM but pronounced H-bonding for MAAAM (Supplementary Figure 6). This intriguing conductance behavior in glycerol, which favors a high-conductance pathway for MAAAM but not for MAAM, is further considered using MD simulations and tICA analysis (*vide infra*). Overall, our results indicate that the choice of solvent stabilizes or disrupts intramolecular H-bonding networks that are relevant for junction formation.

**Figure 2:**
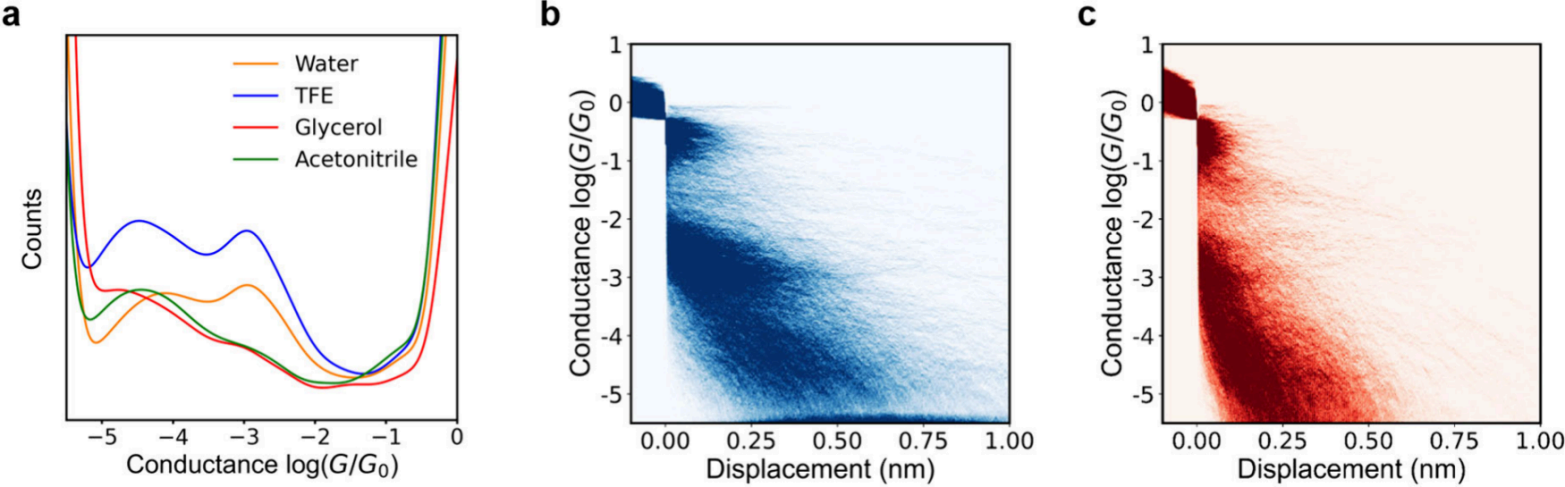
Single-molecule electronic measurements for MAAM in different solvents. (a) 1D conductance histograms for tetrapeptide MAAM in water, 2,2,2-trifluoroethanol (TFE), acetonitrile (ACN), and glycerol. (b) 2D conductance histogram for MAAM in TFE. (c) 2D conductance histogram for MAAM in glycerol. The conductance (*G*) of molecules is reported in units of the conductance quantum, *G*_0_ = 2e^2^/*h*≈77.5 μS.

The high- and low-conductance peak positions exhibit only modest solvent-dependent shifts (Figure 2a, Supplementary Figure 9a, and Supplementary Tables 2,3). In contrast, the corresponding 2D conductance–displacement histograms (Figures 2b,**c** and Supplementary Figures 8 and 9b–d) reveal substantial differences in the displacement range and persistence of these features. These results indicate that solvent effects primarily influence the population distribution and stability of transport-active conformations rather than the intrinsic conductance of a given state. By combining solvent-dependent STM-BJ measurements with molecular dynamics simulations and H-bonding analysis (*vide infra)*, our results show that different solvents selectively stabilize or disrupt intramolecular H-bonding motifs that control access to compact, junction-compatible conformations. In this sense, the solvent does not merely toggle conductance states but actively shapes the conformational landscape through its H-bonding capability.

Single-molecule data were quantitatively analyzed using ML algorithms to classify molecular charge transport behavior into characteristic groups and to identify underlying structure–property relationships (Supporting Information Section S1).^42–45^ Bimodal conductance distributions can arise due to conformationally distinct molecular subpopulations that cannot interchange at equilibrium or during molecular pulling events (static heterogeneity) or due to conformation-dependent conductance states that occur during molecular pulling events (dynamic heterogeneity).^11^ Prior work demonstrated that peptides such as MAAM and MAAAM in water undergo conformation mediated electron transport due to dynamic heterogeneity.^11^ Here, we use silhouette clustering^46^ to determine the optimal number of clusters for data sets corresponding to molecular ensembles for peptides in various solvents including TFE, ACN, and glycerol (Supplementary Figure 10). Results from silhouette clustering indicate that the optimal number of clusters for peptides in various solvents is two. Gaussian mixture modeling (GMM) is further used to analyze the two different clusters identified by silhouette clustering (Supplementary Figures 11 and 12). Results from GMM analysis indicate that Cluster 1 accounts for 5–20% of the single-molecule traces and represents traces in which no molecule is detected, whereas Cluster 2 accounts for 80–95% of the single-molecule traces and exhibits both characteristic conductance populations appearing together in the same traces, consistent with dynamic heterogeneity. From this view, unsupervised ML analysis based on GMM suggests that peptides exhibit conformation-dependent electron transport in various solvents. Although circular dichroism (CD) and solution-phase NMR provide complementary information on bulk, ensemble-averaged conformational behavior, they do not directly resolve transient H-bonding geometries relevant to molecular-scale transport. Nuclear Overhauser effect spectroscopy (NOESY) NMR^47,48^ was employed to probe through-space interactions between protons in MAAM and MAAAM. NOESY NMR shows no persistent through-space contacts (Supplementary Figure 13), consistent with dynamic ensembles, highlighting the need for MD simulations to capture solvent-dependent hydrogen-bonding interactions at the molecular level under nonequilibrium conditions.

To further understand the influence of solvent environments on peptide conformations and electron transport, we performed MD simulations for MAAM and MAAAM in various solvents. In order to mimic the STM-BJ setup, custom potentials were introduced to incorporate the effects of the electric field, the pulling force of the electrode, and the orientation of peptide relative to the electrode surface, as described in prior work (Supporting Information Section S1).^11^ In water, the formation of well-defined secondary structures (β turns or 3_10_ helices) occurs at small molecular junction distances (<6 Å). As the peptides are stretched to larger extensions in the STM-BJ setup, intramolecular H-bonding is abolished (Supplementary Figure 14). However, it is critical to understand the role of H-bonding interactions on peptide conformations in different solvents such as TFE, ACN, and glycerol when characterizing electron transport in single-molecule junctions. Snapshots of MAAM and MAAAM (Supplementary Figures 15 and 16) in various solvents indicate that H-bond-mediated electron transport can occur at short end-to-end distances (∼6 Å), but these interactions are disrupted upon elongation to longer separations (∼12 Å). Importantly, the presence of intramolecular H-bonding alone is not sufficient to yield a pronounced high-conductance state in STM-BJ experiments. Rather, the emergence of well-defined conductance features depends on the probability (*vide infra*) with which such transport-active configurations are sampled under junction conditions.

We performed MD simulations and analyses at small molecular extensions of 6 Å in various solvents including water, glycerol, TFE, and ACN. For MAAM in water, a canonical secondary structure forms at small end-to-end distances, indicative of a β turn (Supplementary Figure 14), which is defined by an H-bond between the carbonyl oxygen of residue *i* and the amide hydrogen of residue *i+3*.^49^ In TFE, a significant fraction of the sampled conformations form secondary structures (Figure 3a), as indicated by the H-bonded population peak at a distance of 2.1 Å, which is less than the threshold distance of H-bond formation (3.5 Å). In contrast, the population of turn-like secondary-structure motifs is strongly reduced in ACN (Figure 3b) and significantly diminished in glycerol (Figure 3c). Given the tetrapeptide length, secondary structure is defined here operationally by the formation of local backbone H-bonds between residues *i* and *i+3*, corresponding to β-turn (or 3_10_-helix-like) conformations. The probabilities of forming these motifs are quantified by calculating the area under the curve of the corresponding distance distributions (0 < *d* < 3.5 Å). The resulting trends are consistent with the single-molecule electronic measurements (Figure 3d). Overall, MD simulations are consistent with the bimodal conductance distributions observed in STM-BJ experiments for MAAM in water and TFE, and the lack thereof in glycerol and acetonitrile, suggesting that the solvent environment is crucial for the electronic properties of peptides.

**Figure 3:**
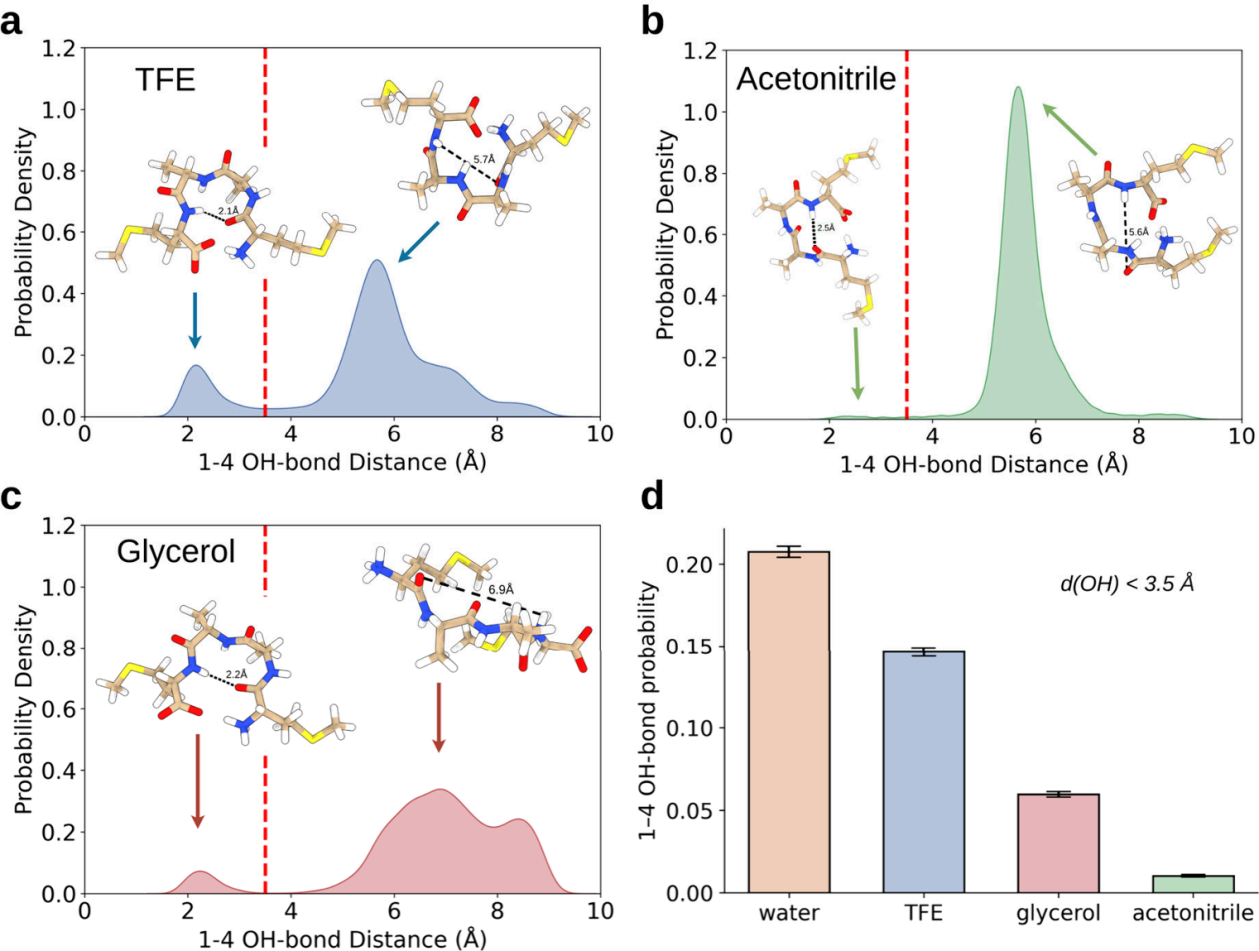
Oxygen–hydrogen bond distance distributions for tetrapeptide MAAM in different solvents including TFE, acetonitrile (ACN), and glycerol. 1D kernel density estimation (KDE) plot for 1 → 4 oxygen–hydrogen bond distances for MAAM in (a) TFE, (b) ACN, and (c) glycerol. (d) Probabilities for forming 1 → 4 oxygen–hydrogen bond in various solvents. Error bars are derived from 100 bootstrapped samples from 50% of the simulation data. The criterion used for bond cutoff is 0.35 nm.

In addition to visualizing the 1D distributions for the H-bond distances, we also analyzed the conformational diversity of peptides by plotting the 2D free energy landscapes. These free energy landscapes were projected on independent components derived from time-lagged independent component analysis (tICA),^50^ a dimensionality reduction technique commonly used in the analysis of molecular dynamics (MD) simulations^47–49^ (Supporting Information Section S1).^51–53^ tICA is used to identify linear combinations of input features such as interatomic distances or dihedral angles that capture the slowest dynamical processes in a system. Unlike traditional methods such as principal component analysis (PCA), which captures directions of maximal variance, tICA focuses specifically on maximizing the autocorrelation of projected features over a specified lag time. This makes tICA particularly suited for systems where slow conformational changes are of interest, such as peptide conformational rearrangement or protein folding.^54,55^

Backbone dihedral angles were used as input features for tICA. The free energy landscapes for MAAM in various solvents, projected onto the first two tICA dimensions, are shown in Figure 4. Free energy landscape for MAAM in TFE (Figure 4a) indicates a deep (high probability) potential well and two shallower minima, which is consistent with the 1D probability distribution observed for TFE showing a peak at 5.7 Å and two additional peaks at 2.1 Å and 7 Å (Figure 3a). We posit that the three minima in the free energy landscape reflect the most probable H-bond distances, as indicated by the peaks in the corresponding oxygen–hydrogen bond distance distributions (Figure 3a). To assess this hypothesis, we mapped the free energy landscape with the H-bond distance (Figure 4b). The color of the minima in this 2D landscape correlates with the peak values observed in the 1D H-bond distribution (Figure 3a). Two dashed boxes are shown in the free energy landscapes, highlighting peptide conformations within and outside the H-bond threshold. It should be noted that the free energy landscape separates the minima according to the presence of secondary structure even though the input to tICA does not include the H-bond distance as a feature. Overall, results from tICA show that H-bond distance, a proxy for secondary structure formation, is the rate determining process for conformational dynamics in MAAM in TFE.

**Figure 4:**
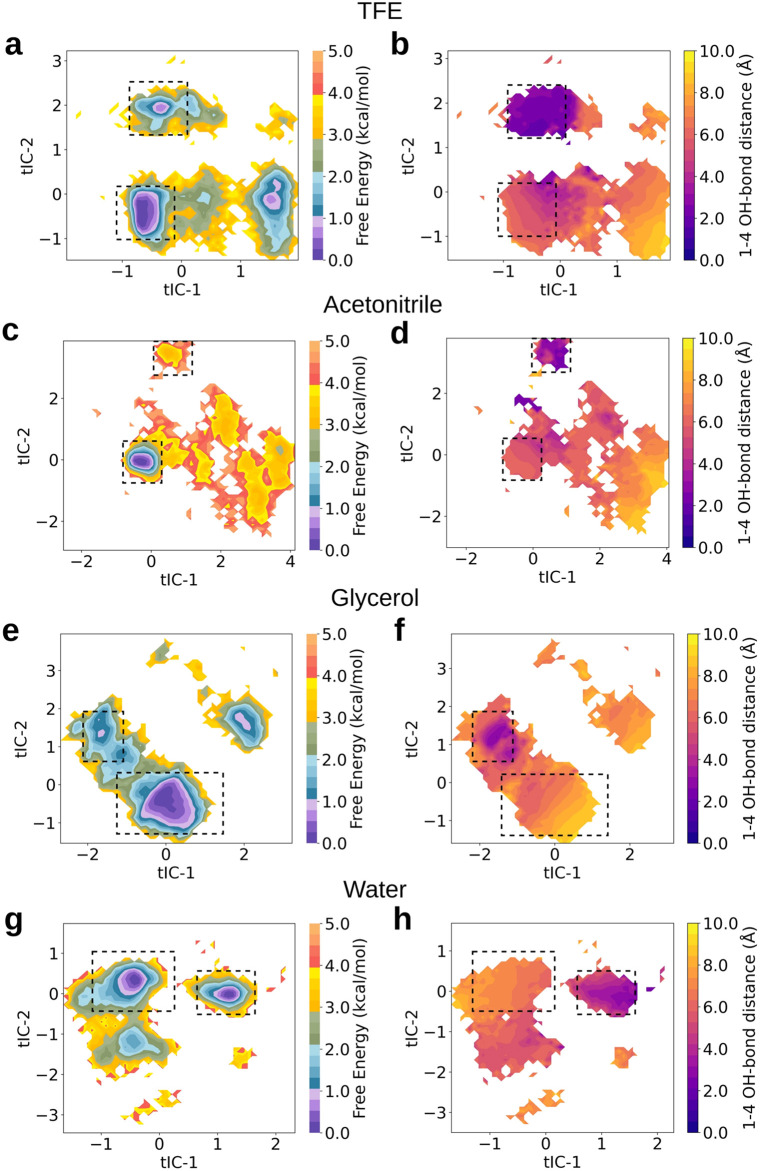
Time-lagged independent component analysis (tICA) of peptides. Free energy landscapes (FEL) for MAAM in various solvents projected on tIC-1 and tIC-2. (a) FELs for MAAM in TFE. (b) FEL mapped by 1–4 oxygen–hydrogen bond distance in TFE. (c) FEL for MAAM in ACN. (d) FEL mapped by 1–4 oxygen–hydrogen bond distance in ACN. (e) FEL for MAAM in glycerol. (f) FEL mapped by 1–4 oxygen–hydrogen bond distance in glycerol.

We next performed MD simulations and tICA for MAAM in acetonitrile. Our results show a significant free energy difference (∼3.5 kcal/mol) between the two minima associated with regions exhibiting or lacking clear secondary structures (Figure 4c,d). This large difference in free energy indicates that secondary structure formation is rare for MAAM in ACN, in agreement with results from single-molecule STM-BJ experiments. In contrast, for glycerol, the deep minimum corresponds to a H-bond distance of approximately 7 Å, which is well beyond the typical threshold for backbone H-bonding in peptides (Figures 4e,f). In addition, for glycerol, the two minima have a relatively lower free-energy barrier compared to ACN, suggesting a greater tendency for transient H-bond formation. This observation is consistent with the 1D probability distribution of H-bond distances for glycerol (Figure 3c), where the peak at 2.2 Å is more pronounced than in ACN, although smaller in magnitude than the corresponding peak observed for TFE, which is consistent with results from single-molecule electronic measurements (Figure 2 and Supplementary Figure 8). tICA results for MAAM in water (Figure 4g,h) are in agreement with the observed results from MD simulations (Supplementary Figure 14) and single molecule experiments (Supplementary Figure 7), consistent with prior work.^11^ The free energy landscapes projected onto the backbone dihedral angles were determined in all solvents (Supplementary Figure 17), illustrating the conformational preferences in different solvent environments. Ramachandran analysis (Supplementary Figure 17) illustrates that TFE and acetonitrile preferentially populate locally compact backbone geometries, including turn-like and α-helical regions, whereas glycerol stabilizes β-sheet and polyproline II-like conformations associated with locally extended backbones. Analysis of the radius of gyration (*R*_*g*_) shows that TFE and acetonitrile favor more compact peptide conformations relative to glycerol, with water exhibiting intermediate behavior (Supplementary Figures 18). It should be noted that global chain compaction does not necessarily correspond to stabilization of specific secondary-structure motifs. Acetonitrile promotes chain compaction (Supplementary Figure 17,18 and Figure 3b) but shows a low probability of intrapeptide 1→4 H-bond formation, consistent with its poor H-bond donor capability and weak acceptor strength (Supplementary Table 1). TFE promotes both compaction and intrapeptide H-bonding (Supplementary Figures 17, and 18 and Figure 3a), reflecting its well-known ability to stabilize turn-like or helical conformations by reducing solvent competition. Glycerol, despite favoring extended conformations, also exhibits a low probability of intrapeptide H-bonding due to strong peptide-solvent H-bonding, which competes effectively with intrapeptide interactions (Supplementary Figures 17,18 and Figure 3c). Overall, our results indicate that solvent-dependent H-bonding competition governs whether compact conformations are supported by intrapeptide H-bonds, as in TFE, or arise from nonspecific collapse or extended ensembles, as in acetonitrile and glycerol.

In performing MD simulations of molecular processes, it is essential to obtain multiple independent simulation replicas to claim ensemble convergence and statistical certainty of key observables. To confirm adequate sampling was achieved in MD simulations, we show that the free energy landscapes remain consistent when constructed from randomly sampled subsets of the simulation data, demonstrating convergence across all systems (Supplementary Figure 19). Overall, these results suggest that the features observed in the MD simulations are not artifacts of insufficient sampling but rather correspond to the experimental pulling process observed during STM-BJ, which occurs as an equilibrium process over the time scales of the experiment (∼100 ms).

We next performed MD simulations of the pentapeptide MAAAM in various solvents. Unlike the tetrapeptide MAAM, the pentapeptide system allows for multiple H-bond mediated electron transport pathways (1→4, 1→5, and 2→5 OH-bonds), which can promote secondary structure formation beyond the 1→4 interaction present in tetrapeptides. Oxygen–hydrogen bond distance histograms (Supplementary Figures 20–23), Ramachandran dihedral free energy landscapes (Supplementary Figure 24), and tICA free energy landscapes (Supplementary Figures 25–28) were determined in several different solvents. Our results suggest complex conformational dynamics for MAAAM in various solvents that occur during the STM-BJ pulling events. Mapping the three potential H-bond distances (1 → 4, 1 → 5, and 2 → 5) onto the free energy landscapes does not reveal a consistent trend across solvents regarding which H-bonding interaction dominates conformational changes and, by extension, electron transport.

In order to understand the complex conformational dynamics of MAAAM in various solvents, we computed the Pearson correlation coefficients (Supplementary Figure 29) between various OH-bond distances (1 → 4, 1 → 5, and 2 → 5) and tIC-1. Our simulation methodology and sampling strategy suggest that the first tIC accurately captures the rate-determining process (Supplementary Figures 14,23). Therefore, the most relevant distance among the 1 → 4, 1 → 5, and 2 → 5 OH bonds is expected to exhibit the strongest correlation. Our results show that tIC-1 correlates most strongly with the 2 → 5 H-bonding interactions in TFE, water and glycerol, and with the 1 → 5 interaction in acetonitrile (Supplementary Figure 29). It should be noted that althoguh tIC-1 (the slowest dynamical mode) is dominated by the 2 → 5 interaction for MAAAM in most solvents, the 1 → 4 H-bond exhibits a significant correlation with tIC-2 (the second slowest mode), particularly in TFE (PCC = 0.45) and glycerol (PCC = 0.54). Overall, these results indicate that 1 → 4 interactions are less stable or less relevant to the dominant electron-transport pathways for MAAAM. Based on the corresponding distance distributions of MAAAM in various solvents (Supplementary Figures 20–23), we infer that TFE, water, and glycerol promote secondary structure formation and, consequently, exhibit a prominent high-conductance state during STM-BJ experiments, consistent with results from CD experiments. In contrast, ACN results in a low probability of the 1→5 interaction (Supplementary Figure 21), suggesting that this solvent does not facilitate similar H-bonding peptide conformations and therefore does not support electron transport across folded peptide conformations like TFE, water, or glycerol. Overall, Pearson correlation coefficients highlight the importance of pathway of electron transport for various solvents.

To further elucidate the origin of the distinct molecular-scale electronic signatures observed for MAAM and MAAAM in glycerol, we analyzed the end-to-end distance distributions obtained from all-atom MD simulations. In glycerol, MAAAM samples a substantially broader conformational space than MAAM, whereas acetonitrile collapses both peptides to short end-to-end distances and TFE stabilizes discrete bimodal populations (Supplementary Figure 30). To further examine the mechanistic differences between MAAM and MAAAM in glycerol (Supplementary Section S8), we computed the coefficient of variation for MAAAM relative to MAAM across different solvents. We observe an 81% increase in the coefficient of variation in glycerol, which is significantly larger than in the other solvents studied here (Supplementary Figure 31). These results indicate that glycerol provides a permissive solvation environment in which intrinsic backbone flexibility dominates conformational sampling, enabling MAAAM to transiently access compact, junction-compatible geometries that the more conformationally constrained MAAM cannot readily sample. Overall, the combination of MD simulations, tICA analysis, and Pearson correlation coefficients provides a molecular-level framework for interpreting the single-molecule experimental results.

In this work, we investigate the electronic properties of the peptides MAAM and MAAAM in various solvents including water, acetonitrile, glycerol, and 2,2,2-trifluoroethanol, using a combination of experiments and computational modeling. Single-molecule electronic experiments reveal that peptides exhibit a bimodal conductance distribution in water and TFE, where a high-conductance state linked to a defined secondary structure (β turn or 3_10_ helices) and a low-conductance state associated with primary structures. The electron-transport pathway associated with the secondary structure is markedly diminished in acetonitrile, consistent with its polar aprotic character and its tendency to denature or weakly stabilize hierarchical structures. Surprisingly, glycerol suppresses the high-conductance state in MAAM but not in MAAAM, underscoring the nuanced coupling between solvent environment and the structural motifs of closely related peptide systems. MD simulations, time-lagged independent component analysis (tlCA), and Pearson correlation coefficients are used to rationalize experimental observed results. Results from MD simulations and tlCA of intramolecular H-bonding distances across different solvents reveal a peak associated with dominant H-bonding interactions, which corresponds to the experimentally observed high-conductance state and supports the experimental findings. Pearson correlation coefficients are further used to understand the electron transport behavior in different solvents. Overall, our results demonstrate that solvent-dependent stabilization or disruption of intramolecular H-bonding motifs actively shape the conformational landscape of peptides and thereby governs access to compact, junction-compatible structures that dominate single-molecule electron transport. Our results indicate that solvents primarily modulate the stability and accessibility of transport-active (compact) conformations under junction elongation. From this perspective, our work reveals that the solvent environment plays a critical role in controlling peptide conformation and the resulting electron-transport characteristics. The insights gained from this study provide a framework for understanding how solvent environments influence electron transport in longer peptides and more complex protein structures. Future studies may further explore the role of intermolecular interactions and supramolecular assembly^56–58^ in biomolecular electron transport, particularly in systems specifically designed to stabilize intermolecular transport-active configurations.

## Supplementary Material



## Data Availability

The scripts used for molecular dynamics (MD) simulations, analysis, as well as the featurized trajectories used in this work for reference to reproduce results in the future are available at: https://github.com/ShuklaGroup/Solvent_electron_transport_2025

## Notes

Additional experiments and computational details, including characterization data such as mass spectrometry and circular dichroism, additional single-molecule conductance measurements, molecular dynamics simulations, two-dimensional (2D) nuclear magnetic resonance (NMR) spectroscopy, and unsupervised machine learning results based on Gaussian mixture modeling (GMM) (PDF)
