# Supplementary material for: Solvent Environment Influences Molecular Conformation and Electron Transport in Peptides
Source: J Phys Chem Lett. 2026 May 17;17(21):6004–13. doi: 10.1021/acs.jpclett.6c01257 (PMC13250990; doi:10.1021/acs.jpclett.6c01257)
Supplement: Supplementary file 1 [file jz6c01257_si_001.pdf]

Supplementary Information for:

# Solvent environment influences molecular conformation and electron transport in peptides


Rajarshi Samajdar[1,2†], Hassan Nadeem[2,3†], Neil Moghe[1], Diwakar Shukla[1,3,4,6*], Charles M. Schroeder[1,2,4,5,6*‡]

[1]Department of Chemical and Biomolecular Engineering, University of Illinois Urbana-Champaign, Urbana, IL 61801, United States
[2]Beckman Institute for Advanced Science and Technology, University of Illinois Urbana-Champaign, Urbana, IL, 61801, United States
[3]Department of Bioengineering, University of Illinois Urbana-Champaign, Urbana, IL, 61801, United States
[4]Center for Biophysics and Quantitative Biology, University of Illinois Urbana-Champaign, Urbana, IL, 61801, United States
[5]Department of Materials Science and Engineering, University of Illinois Urbana-Champaign, Urbana, IL, 61801, United States
[6]Department of Chemistry, University of Illinois Urbana-Champaign, Urbana, IL, 61801, United States

[†]These authors contributed equally

*Corresponding authors. Email: diwakar@illinois.edu, cschroeder@princeton.edu

[‡]C.M.S.: Present address: Department of Chemical and Biological Engineering, Princeton University, 35 Ivy Lane, Princeton, NJ, 08540, United States

**Contents**

S1. General methods ……………………………………………………………… 3

S2. Mass spectrometry ………………………………………………………… 8

S3. Circular dichroism (CD) data …………………………………………………. 10

S4. Single-molecule charge transport experiments ……………………………….. 14

S5. Silhouette clustering and Gaussian mixture modeling (GMM) ……..………… 18

S6. Two-dimensional (2D) nuclear magnetic resonance (NMR) spectroscopy …. 21

S7. Molecular dynamics (MD) simulations ……………………………………….. 22

S8. Glycerol induced changes in conformational accessibility …………………... 36

S9. References ………………………………………………………………… 38

## S1. General methods

### Oligopeptide sequences

Oligopeptides MAAM and MAAAM were purchased from GenScript (Piscataway, NJ). Electrospray ionization (ESI) mass spectrometry data for these peptide sequences are provided below (**Supplementary Figures 1,2**).

### Circular dichroism (CD) experiments

Circular dichroism (CD) measurements were performed using a Jasco J-1500 spectrophotometer in the range of 190 nm to 240 nm at room temperature. CD measurements were carried out for peptides in various solvents—water, acetonitrile, and 2,2,2-trifluoroethanol (TFE). CD measurements were obscured in pure glycerol due to a poor signal-to-noise ratio and/or limited light penetration through the sample. To characterize the influence of glycerol on the secondary structures of MAAM and MAAAM, CD experiments were performed in glycerol-water mixtures with low glycerol content (0.5% glycerol in water).

### Two-dimensional nuclear magnetic resonance (2D NMR)

Nuclear Overhauser effect spectroscopy (NOESY) NMR was performed on Carver B500 Bruker Advance III HD.

### Single-molecule conductance measurements

Single-molecule conductance measurements were performed using a custom-built scanning tunneling microscope break junction (STM-BJ), as previously described.[1–6] Gold STM tips were prepared using 0.25 mm Au wire (99.998%, Alfa Aesar). STM-BJ experiments were performed in Corning cell culture grade water (product number 255-055-CV), acetonitrile (Sigma Aldrich), glycerol (Avantor, Inc.), and 2,2,2-trifluoroethanol (Sigma Aldrich). Due to the polarity of the solvents used in this work, STM tips were coated with an Apiezon wax to prevent Faradaic currents from masking characteristic molecular features.[7] Gold substrates for the measurements were prepared by evaporating 120 nm of gold on polished AFM metal discs (Ted Pella). Peptide concentrations (<1 mM) were selected to yield Poisson statistics in molecular conductance traces. Conductance histograms are generated for all molecules without data selection.

One-dimensional (1D) conductance histograms were used to identify characteristic high- and low-conductance features, whereas two-dimensional (2D) conductance–displacement histograms were employed to assess the displacement range and persistence of these features during junction elongation. The latter provide additional information on the stability and accessibility of transport-active conformations that are not captured by peak position alone.

### Gaussian Mixture modeling (GMM) & silhouette score clustering

Unsupervised learning algorithms (such as K-means++[8] and spectral clustering analysis[9]) have been previously used to analyze multiple conductance populations during single-molecule charge transport experiments. To understand the bimodal conductance distribution observed for peptides, we employ a classification algorithm for data clustering

based on Gaussian mixture modelling (GMM), as described in prior work.[4] GMM offers advantages over alternative methods such as K-means due to its ability to detect sub-populations of unequal covariance.

From each individual trace, a 30-by-30 two-dimensional histogram and a 100-bin one dimensional histogram are extracted. The 30×30 two-dimensional histogram captures the relationship between conductance and displacement, whereas the 100-bin one-dimensional histogram captures the overall conductance distribution for that trace. Combining these into a 1000-dimensional feature space (30×30 + 100) allows each trace to be represented by a comprehensive set of features over which GMM operates. The classification operates on the conductance range of 0 to -5.5 $\log(G/G_o)$, and displacement range of -0.1 nm to 1 nm (0 nm corresponds to the point where the junction is broken, and the -0.1 nm to 0 nm regime indicates metal-metal contact). Here, $G_0 = 2e^2/h \approx 77.5$ µS is the quantum of conductance. Normalizing the conductance of each trace by $G_0$ allows for a dimensionless, physically meaningful scale, enabling direct comparison across different molecular junctions and experiments. Using $\log(G/G_0)$ further aids in analyzing features and identifying clustering patterns in conductance values. All traces are aligned at 0.5 $G_o$ as the starting point of displacement. Silhouette scores[10] are calculated for calculating the number of clusters based on GMM. Silhouette scores indicate how similar a feature is to its own cluster compared to different clusters. Silhouette score value ranges from -1 to 1. For MAAM and MAAAM, the silhouette score values are computed for the number of clusters varying from 2 to 5. The largest value of silhouette score is taken as an indication for optimal number of clusters. The goal of using silhouette scores is to find the optimal number of clusters for our bimodal conductance distribution and to observe if the two-conductance populations are part of a single trace, indicating conformation induced charged transport (dynamic heterogeneity), or they occur in separate traces (static heterogeneity), indicating different native conformations or distinct charge transport pathways.

**Custom potentials for molecular dynamics (MD) simulations**

A key challenge for simulating single-molecule pulling processes is large difference between the pulling rates used in experiments and those accessible by MD simulations. Typical experimental pulling rates are on the order of Angstroms per millisecond (1 Å per 5 ms in present study), whereas single-trajectory MD simulations (at most) typically reach ms timescales, e.g., with the use of bespoke hardware[11] or massively distributed computing schemes[12]. In addition, the need for multiple independent simulation replicas to claim ensemble convergence and statistical certainty of key observables further restricts simulations to sub-experimental timescales. However, because the experimental pulling rate is also slow relative to characteristic relaxation timescales of small peptides, we assume that all molecular conformations accessible at a given end-to-end distance are sampled during each step of the experimental pulling process. In other words, experimental pulling occurs as an equilibrium process. Rather than performing costly simulations of the entire pulling process, it is more computationally feasible to simulate the molecular junction at various holding (end-to-end distance) stages representing the different separation distances arising during the pulling experiments. In this work, MD

simulations are performed at fixed an end-to-end distance corresponding to experimentally relevant junction separations.

Using the above approach, MD simulations can be performed to replicate single molecule break junction experiments as described in prior work[4]. Using this approach, we performed a series of independent simulations where we restrained the end-to-end (sulfur-sulfur) distance along the pulling axis. The functional form of the potential utilized to enforce this restraint is given in Equation 1:

$$U_1 = \frac{1}{2}k_1[(z_{S_2} - z_{S_1}) - z_0]^2, \quad (1)$$

where the coefficient $k_1$ is the force constant of the harmonic potential, $z_{S1}$ and $z_{S2}$ are the *z*-coordinate of the sulfur atoms of the N-terminal and C-terminal methionine residues respectively, and $z_0$ is the equilibrium distance for the given stage. We use a value of 1 kcal/mol/Å$^2$ for $k_1$, and we utilize three independent holding stages with $z_0$ equal to either 6 Å, 9 Å, or 12 Å. This force constant was selected such that the resulting distributions of $z_{S2} - z_{S1}$ distances have slight overlap.

By restraining the *z*-displacement between the sulfur atoms, rather than the distance, the movement of each sulfur atom is effectively restrained to one of two parallel planes which implicitly represent two parallel planes of gold electrode.

A potential is introduced to represent an applied electric field due to the voltage difference across the two electrodes. The functional form is given in Equation 2:

$$U_2 = \sum_{i=1}^{N_{atoms}} -q_i E z_i = \sum_{i=1}^{N_{atoms}} -q_i \left(\frac{V}{z_0 + 2l_{S-Au}}\right) z_i \quad (2)$$

where $N_{atoms}$ is the total number of atoms in each system including solvent, $q_i$ is the charge of atom *i*, $z_i$ is the z-coordinate of atom *i*, $z_0$ is the equilibrium end-to-end distance (displacement along *z*) for a holding stage, and $l_{S-Au}$ is the length of the sulfur-gold bond.

We further introduce a potential to orient each sulfur atom's lone pairs in either the positive or negative *z*-direction, such that a feasible dative bond may occur between the sulfur and a fictitious gold particle. Because electron lone pairs are not explicitly represented in atomistic MD simulations, we define surrogate vectors that involve each sulfur's adjacently bonded carbon atoms to act as a proxy for the direction of the electron lone pairs. We impose a restraint directly on the dot product of each surrogate vector with the pulling axis. The functional form of this potential is shown in Equation 3:

$$U_3 = \sum_{i=1}^{2} k_3 \left[\left|\vec{r}_{S_i} - \left(\frac{\vec{r}_{CG_i} - \vec{r}_{CE_i}}{2}\right)\right| \cdot \vec{z}\right] = \sum_{i=1}^{2} k_3[|\vec{p}_i| \cdot \vec{z}](-1)^i \quad (3)$$

where $\vec{r}_{S_i}$ represents the three-dimensional Cartesian coordinates of the sulfur atom of interest, with $S_1$ and $S_2$ subscripts indicating the identity of the sulfur atoms in the N-terminal and C-terminal methionine residues, respectively, $\vec{r}_{CG_i}$ and $\vec{r}_{CE_i}$ are Cartesian coordinates of the adjacent carbon atoms covalently bonded to each sulfur of interest, and $\vec{z}$ is the unit vector in the direction of the *z*-axis. Vertical lines denote vector normalization. The final term in the equation determines the sign of the potential (and thus the direction of the surrogate vector) allowing for one sulfur's lone pair to be oriented in the positive *z*-direction while the other is oriented oppositely in the negative *z*-direction. The value of $k_3$ is taken as 10 kcal/mol, resulting in a strong potential that tightly secures the orientation of sulfur lone pairs towards the implicitly represented gold electrodes.

**Molecular dynamics (MD) simulations**

MD simulations were performed to generate conformational ensembles for MAAM and MAAAM molecular junctions at an anchor displacement of 6 Å to understand the interplay between hydrogen bonding (H-bonding) and electron transport pathways, as described in prior work.[4] Initial peptide structures were constructed using PeptideBuilder python package.[13] 16 replicates for MAAM and 20 replicates for MAAAM were initialized with randomized dihedral angles. PACKMOL[14] was used to initialize the tetra- and pentapeptide in various solvents (water, acetonitrile, 2,2,2-trifluoroethanol, and glycerol). Peptide structures were solvated in a cubic box of TIP3P water force field.[15] CHARMM additive forcefield was used for glycerol,[16] and CHARMM general forcefield[17] was used for acetonitrile and 2,2,2-trifluoroethanol. PSFGEN[18] plugin in VMD was utilized to create PSF topology files.

MD simulations were performed using OpenMM 7.7.0.[19] A minimization protocol was followed by an NVT ensemble simulation performed for 1 ns. The system was heated from a temperature of 5 K to 300 K. Protein backbone atoms were restrained during the NVT simulation using a spring constant of 1.0 kcal/mol·Å$^2$. An NPT ensemble simulation was then performed for 1.0 ns: the first 0.5 ns with restrained backbone atoms, followed by 0.5 ns without restraints. This was followed by an additional equilibration step using an NPT simulation without restraints for 1.0 ns. Production simulations were performed in the NPT ensemble for 200 ns per replicate, resulting in a total of 3.2 μs of simulation for MAAM and 4.0 μs for MAAAM. A series of custom potentials were implemented to implicitly represent interactions between the peptide and gold particles, replicating the STM-BJ experiments as described in our previous work.[20] The non-bonded interactions were computed using particle mesh Ewald[21] (PME) method with a cutoff of 1.2 nm. Constraints were applied to the hydrogen bonds, and water molecules were kept rigid. A timestep of 2 femtoseconds was employed, with a Langevin thermostat set to 2.8284/picosecond for the friction coefficient. The system was maintained at 1.0 atmosphere of pressure for the NPT ensemble. Simulation frames were saved at an interval of 100 ps for analysis.

**Time-lagged independent component analysis (TICA)**

MDTraj[22] was used to perform structural analysis of the simulation trajectories and feature extraction. All systems were characterized using backbone dihedral angles. Deeptime[23] was used to illustrate free energy landscapes, with 50 bins. Deeptime[23] was also used to

perform time-lagged independent component analysis (tICA).[24] with a lag-time of 1ns. Pearson correlation coefficients[25,26] were then computed between the original feature set and the resulting tICA components to determine which structural features were most strongly represented in the reduced-dimensionality space, in particular tIC-1 (the slowest process).

## S2. Mass spectrometry

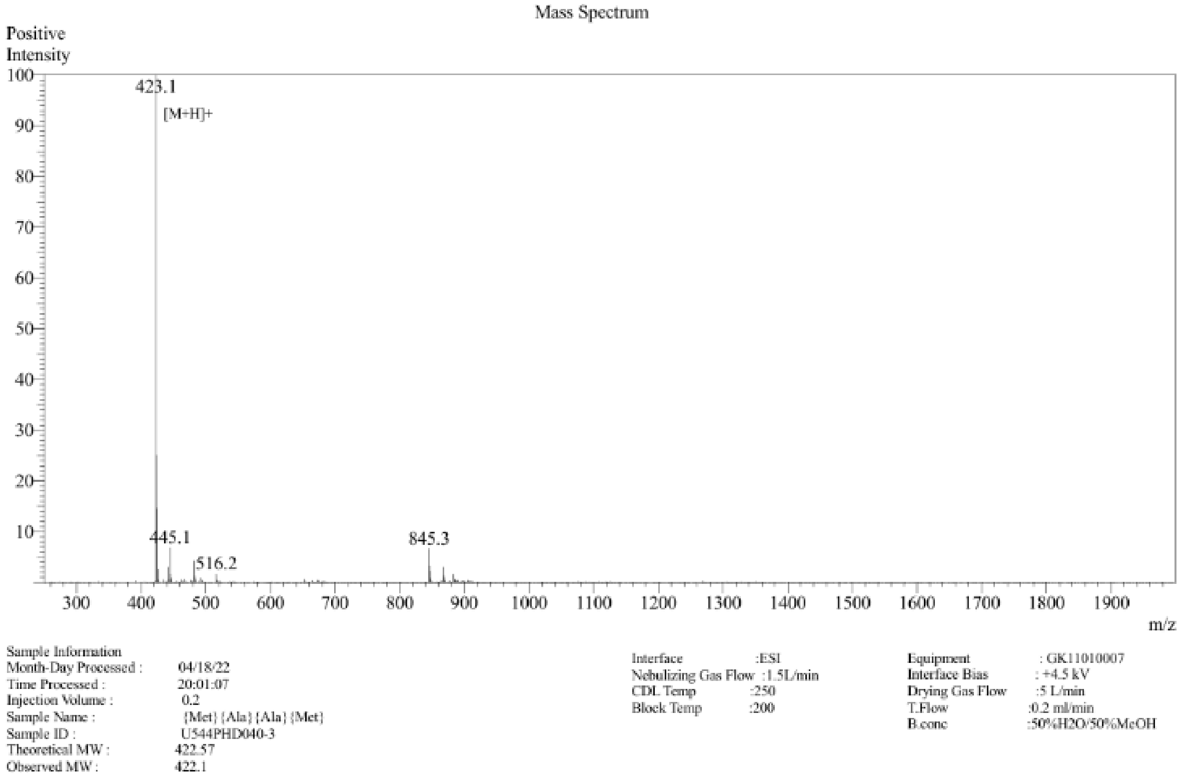


**Supplementary Figure 1**: Electrospray ionization (ESI) mass spectrometry data for peptide sample MAAM. Theoretical molecular weight is 422.57 m/z. Observed molecular weight is 422.1 m/z.

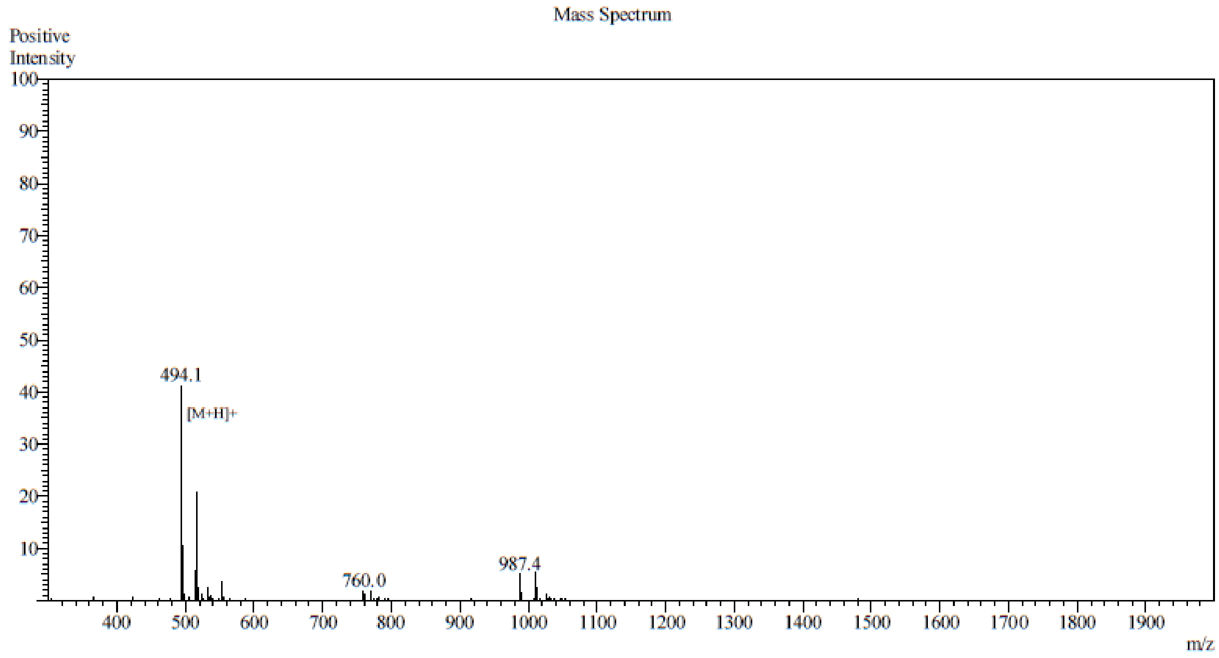


**Supplementary Figure 2**: Electrospray ionization (ESI) mass spectrometry data for peptide sample MAAAM. Theoretical molecular weight is 493.64 m/z. Observed molecular weight is 493.1 m/z.

## S3. Circular Dichroism

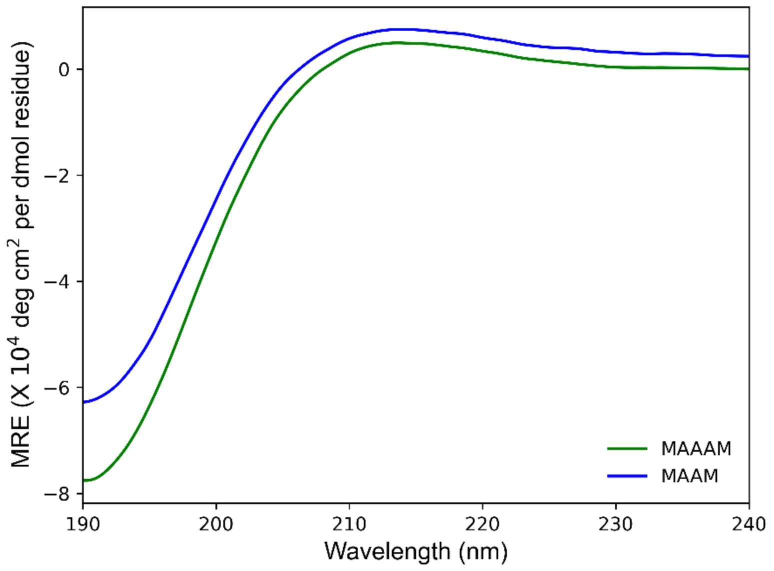


**Supplementary Figure 3**: Circular dichroism (CD) spectra for MAAM and MAAAM in water. The spectral features observed are consistent with prior published work[4].

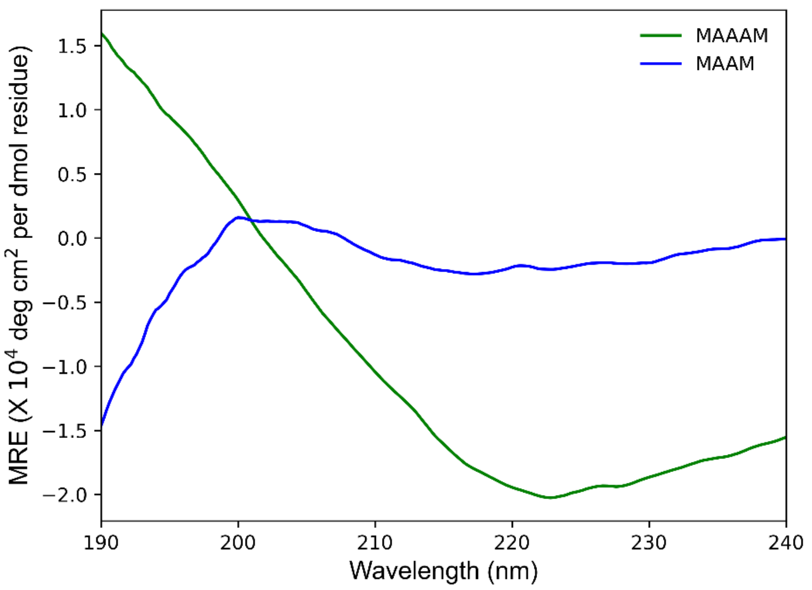


**Supplementary Figure 4**: Circular dichroism (CD) spectra for MAAM and MAAAM in 2,2,2-trifluoroethanol (TFE).

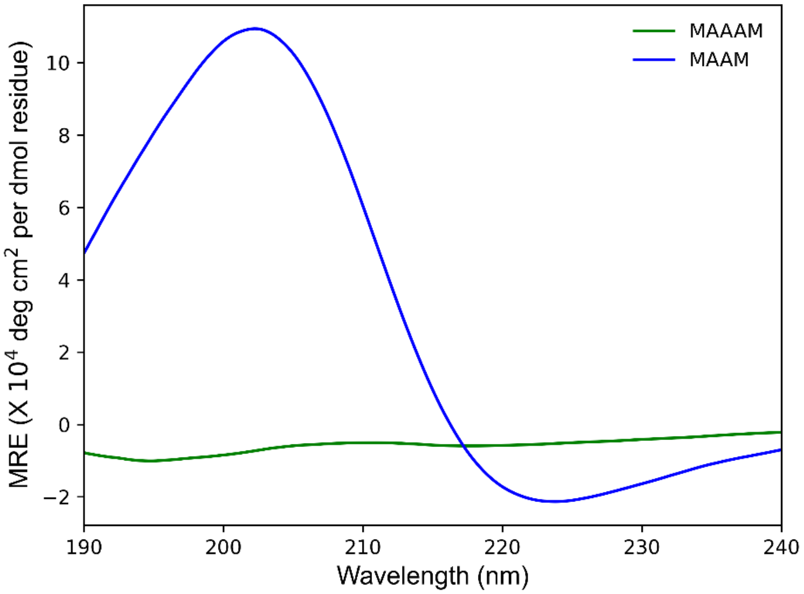


**Supplementary Figure 5**: Circular dichroism (CD) spectra for MAAM and MAAAM in acetonitrile (ACN).

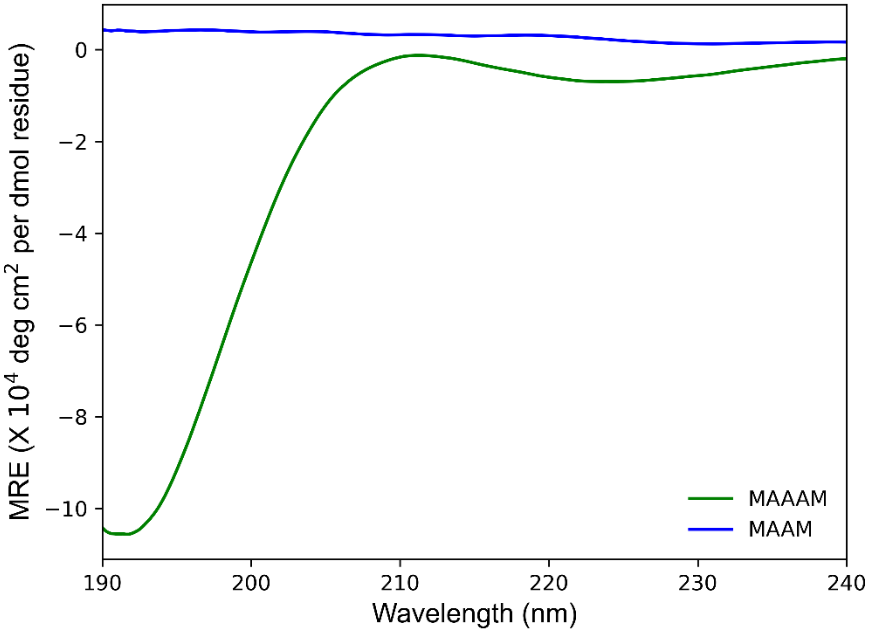


**Supplementary Figure 6**: Circular dichroism (CD) spectra for MAAM and MAAAM in glycerol-water mixture with low glycerol content (0.5% glycerol in water).

## S4. Single-molecule electron transport experiments

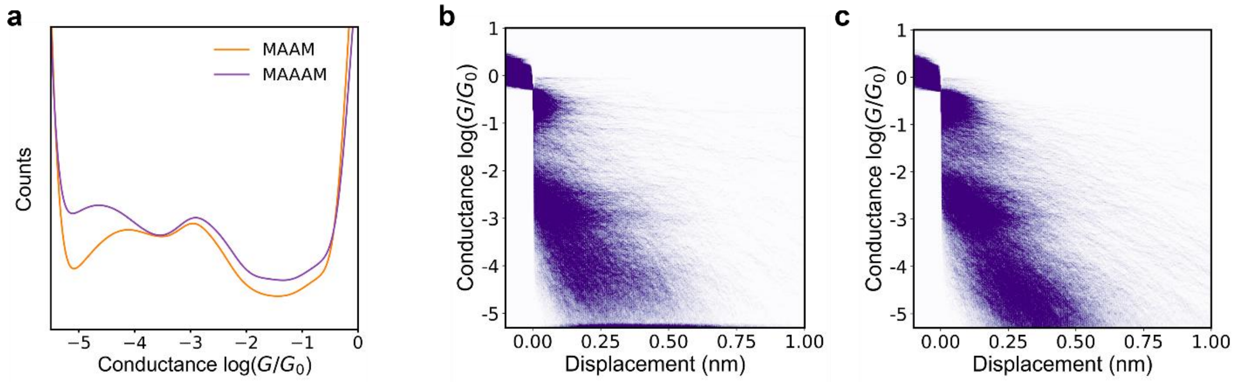


**Supplementary Figure 7**: STM-BJ data for MAAM and MAAAM in water, as reported in our prior work.[4] **(a)** 1D conductance histograms for MAAM and MAAAM. 2D conductance histograms for **(b)** MAAM and **(c)** MAAAM. Data were obtained using 0.1 mM concentrations of peptides at an applied bias of 250 mV across ensembles of >5000 single molecules.

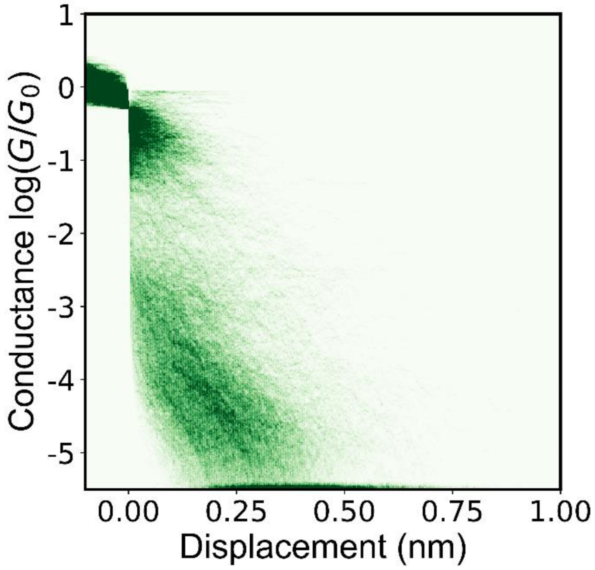


**Supplementary Figure 8**: 2D conductance histogram for MAAM in acetonitrile. Data were obtained using 0.1 mM concentrations of peptides at an applied bias of 250 mV across ensembles of >5000 single molecules.

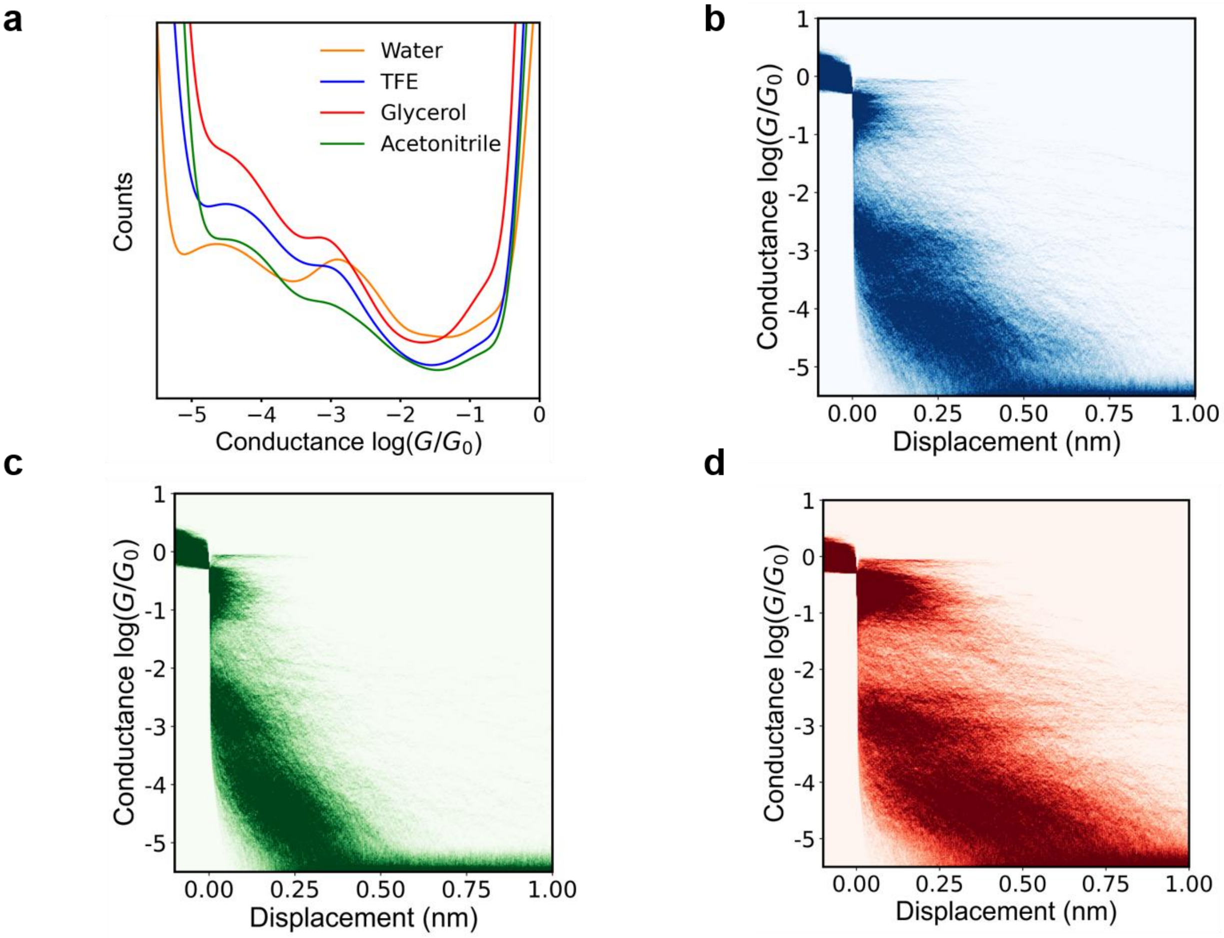


**Supplementary Figure 9**: 1D and 2D conductance histograms for MAAAM in various solvents – water, 2,2,2-trifluoroethanol (TFE), acetonitrile (ACN), and glycerol. Data were obtained using 0.1 mM concentrations of peptides at an applied bias of 250 mV across ensembles of >5000 single molecules.

**Supplementary Table 1:** Unified comparison of solvents variables. Dielectric constants, $E_T(30)$ values, hydrogen-bond donor and acceptor capabilities of all solvents are listed.

| Solvent | Dielectric constant (25 °C) | $E_T(30)$ (kcal/mol) | H-bond Donor | H-bond Acceptor |
|---|---|---|---|---|
| Water | ~78[27] | ~63[28] | Strong | Strong |
| 2,2,2-trifluoroethanol (TFE) | ~27[29] | ~60[28,29] | Strong | Weak |
| Acetonitrile (ACN) | ~36[30] | ~45[28,30] | None | Weak |
| Glycerol | ~42[31] | 57[32] | Strong | Strong |

**Supplementary Table 2:** High conductance and low conductance peaks for the MAAM in various solvents. The conductance value, in log scale, for each peak (high conductance and low conductance) is determined from the center position of a Lorentzian fit to the peak. MAAM in glycerol and acetonitrile does not have a well-defined high conductance peak.

| **MAAM peptide** | **High conductance peak** [log($G/G_0$)] | **Low conductance peak** [log($G/G_0$)] |
|---|---|---|
| Water | -2.86 | -4.22 |
| 2,2,2-trifluoroethanol (TFE) | -2.88 | -4.40 |
| Acetonitrile (ACN) | -2.90 | -4.35 |
| Glycerol | -2.87 | -4.48 |

**Supplementary Table 3:** High conductance and low conductance peaks for the MAAAM in various solvents. The conductance value, in log scale, for each peak (high conductance and low conductance) is determined from the center position of a Lorentzian fit to the peak. MAAM in acetonitrile does not have a well-defined high conductance peak.

| **MAAAM peptide** | **High conductance peak** [log($G/G_0$)] | **Low conductance peak** [log($G/G_0$)] |
|---|---|---|
| Water | -2.91 | -4.63 |
| 2,2,2-trifluoroethanol (TFE) | -2.98 | -4.48 |
| Acetonitrile (ACN) | -2.92 | -4.45 |
| Glycerol | -3.02 | -4.43 |

## S5. Silhouette Clustering and Gaussian Mixture Modeling (GMM)

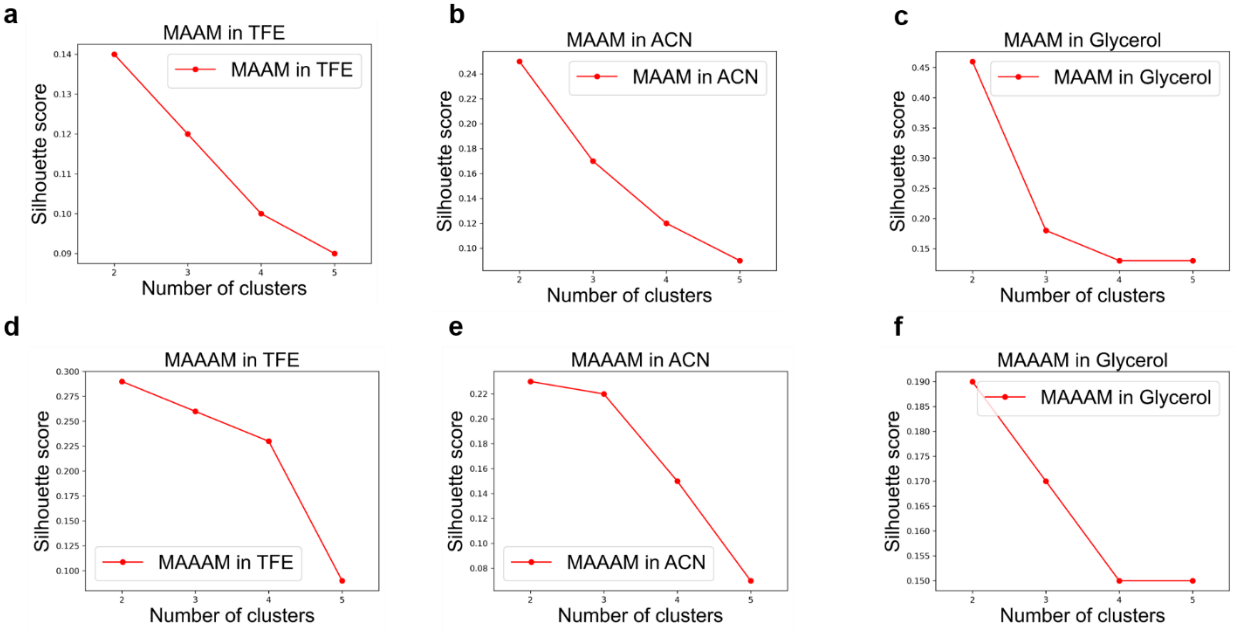


**Supplementary Figure 10**: Silhouette scores for MAAM and MAAAM in various solvents indicating that the number of clusters is two.

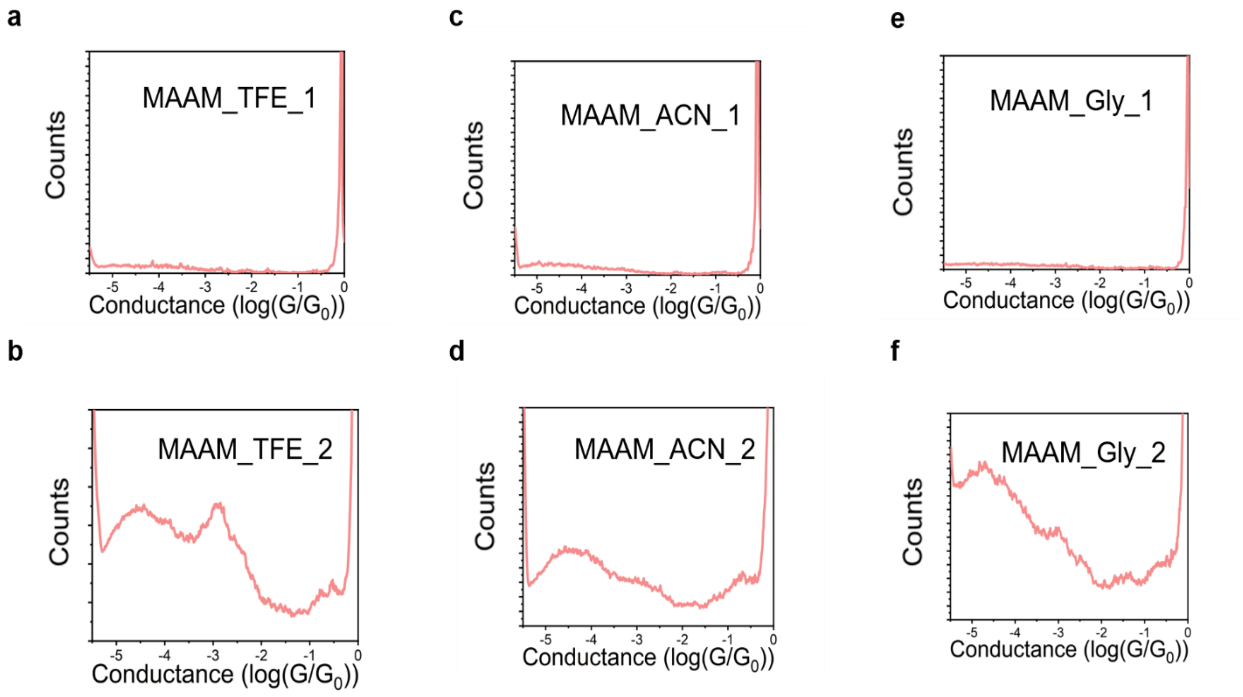


**Supplementary Figure 11**: Gaussian mixture modeling (GMM) for MAAM in TFE, acetonitrile, and glycerol. Cluster 1 accounts for only 5-20% of the data and represents traces in which no molecule is detected or only background signal is observed. Cluster 2 accounts for 80-95% of the single molecule traces and shows both characteristic conductance populations appearing together in the same molecular traces indicating conformation mediated electron transport (dynamic heterogeneity).

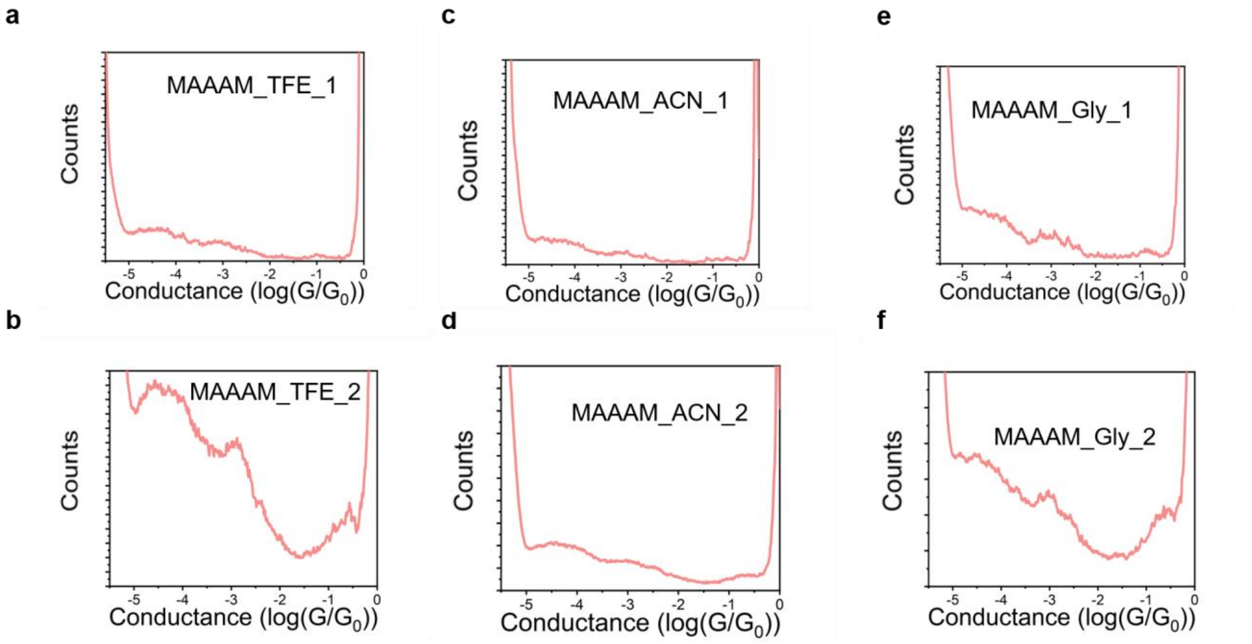


**Supplementary Figure 12**: Gaussian mixture modeling (GMM) for MAAAM in TFE, Acetonitrile, and glycerol. Cluster 1 accounts for only 5-20% of the data and represents traces in which no molecule is detected or only background signal is observed. Cluster 1 accounts for 80-95% of the single-molecule traces and shows both characteristic conductance populations appearing together in the same molecular traces. Cluster 1 traces exhibit substantially lower counts and are dominated by background or weakly formed junctions, and occasional bimodal features reflect sporadic sampling of conductance states that lack the statistical weight observed in Cluster 2.

## S6. Two-dimensional (2D) nuclear magnetic resonance (NMR) spectroscopy

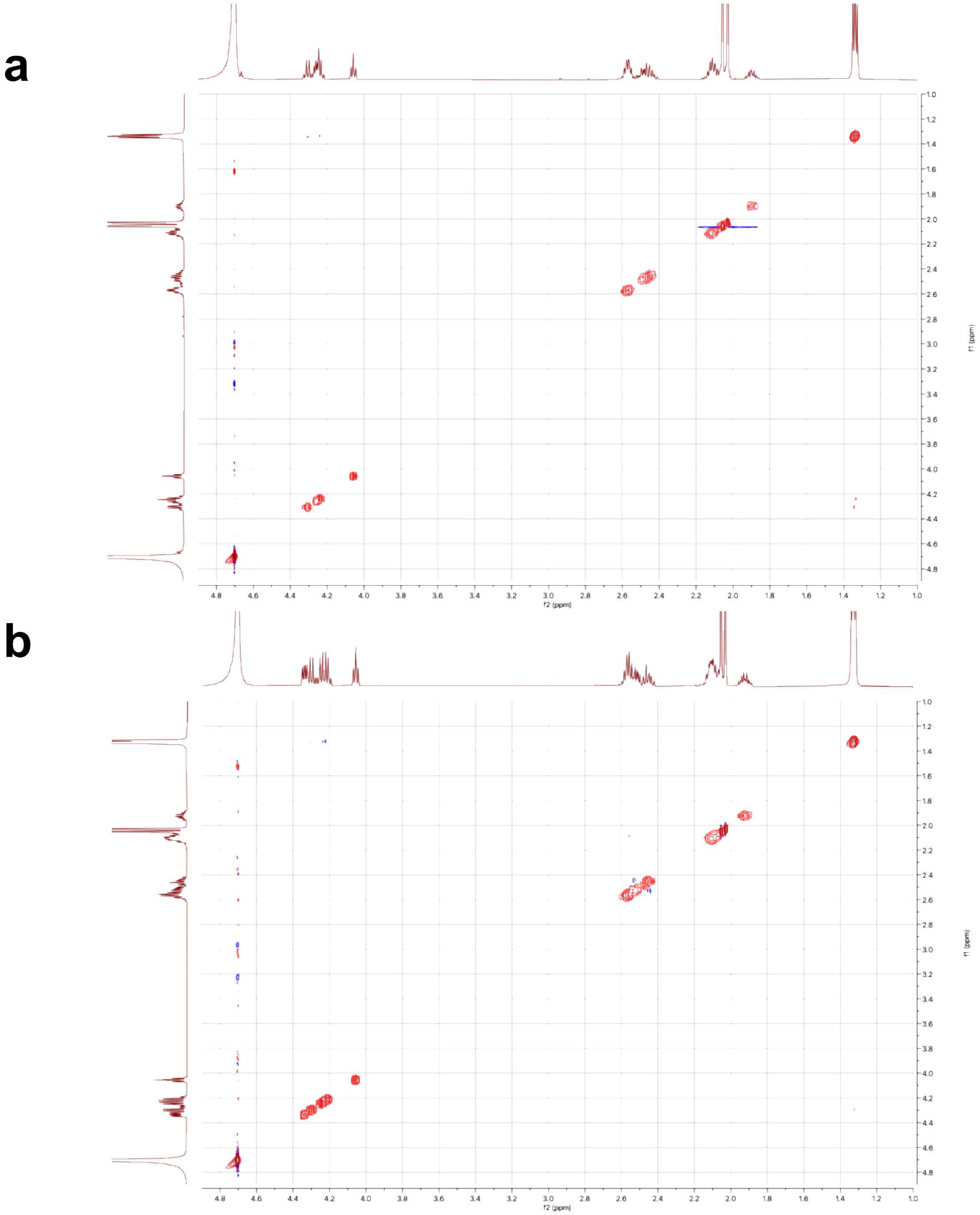


**Supplementary Figure 13:** Nuclear Overhauser Effect Spectroscopy (NOESY) NMR for (a) MAAM and (b) MAAAM. Deuterium oxide ($D_2O$) was used as the solvent. The results suggest lack of close proton–proton spatial contacts in solution phase.

## S7. Molecular dynamics (MD) simulations

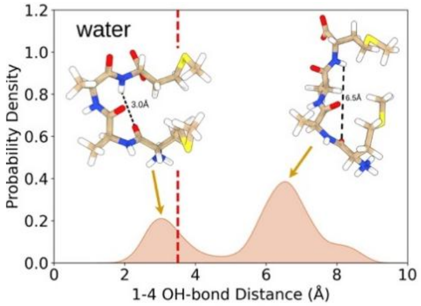


**Supplementary Figure 14**: 1D kernel density estimation (KDE) plot for 1→4 OH distance in MAAM for water along with representative conformers for each peak. These results are consistent with prior published results[4] for MAAM in water.

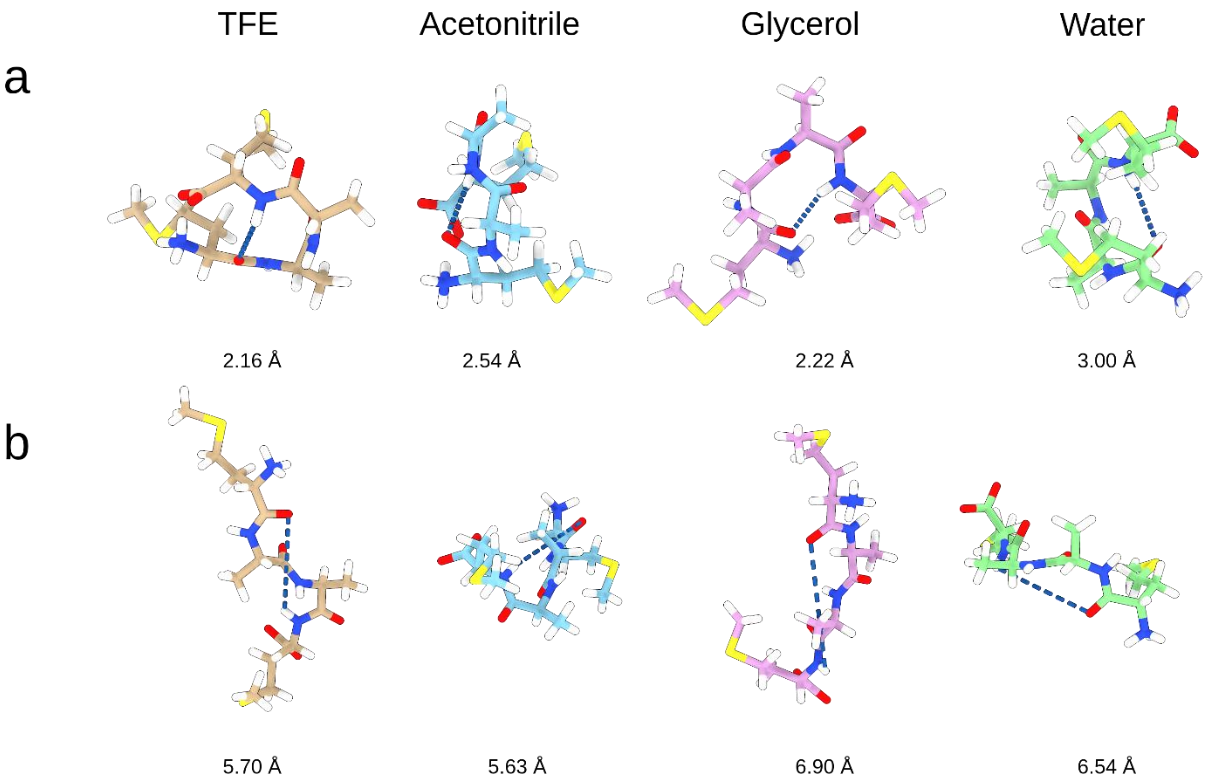


**Supplementary Figure 15**: Snapshots of MAAM in various solvents showing **(a)** folded (1→4 H-bonding) and **(b)** extended peptide conformations corresponding to the high- and low-conductance states, respectively, as observed in our single-molecule electronic experiments.

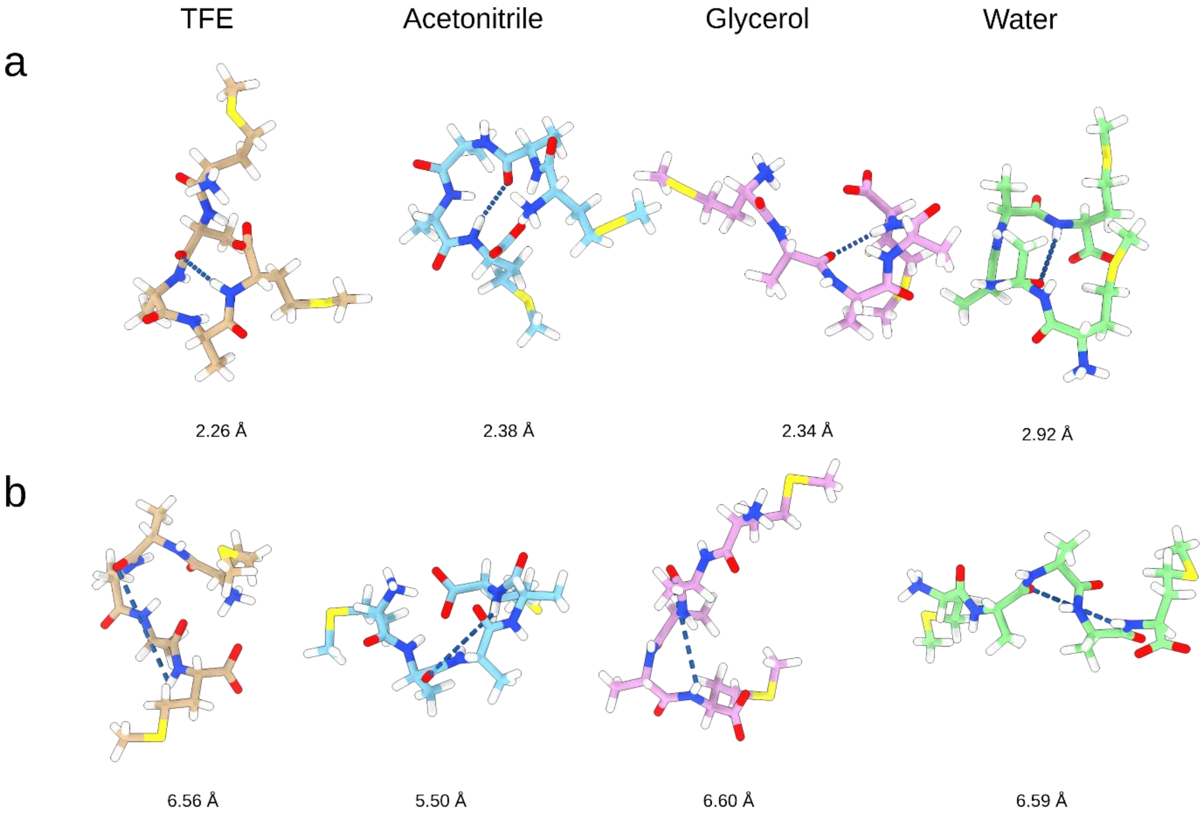


**Supplementary Figure 16**: Snapshots of MAAM in various solvents showing **(a)** folded and **(b)** extended peptide conformations corresponding to the high- and low-conductance states, respectively, as observed in our single-molecule electronic experiments.

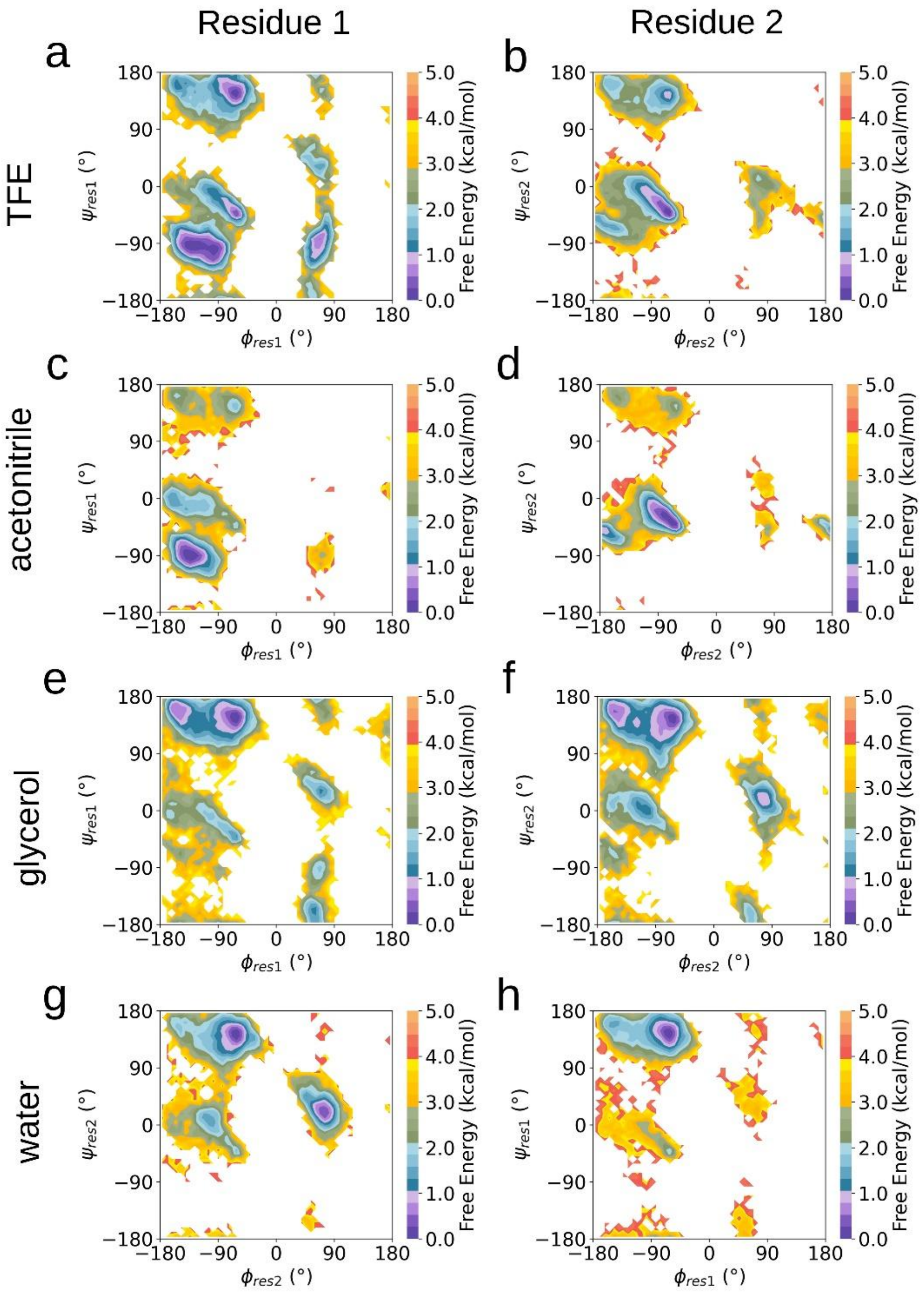


**Supplementary Figure 17**: Ramachandran dihedral free energy landscapes (FELs) for MAAM tetrapeptide in various solvents. Residues 1 and 2 refer to the first and second central alanine residues respectively. **(a),(b)** Dihedral FEL for residue 1 and 2 in 2,2,2-trifluoroethanol TFE. (**c),(d)** Dihedral FEL for residue 1 and 2 in acetonitrile (ACN). (**e),(f)** Dihedral FEL for residue 1 and 2 in glycerol. **(g)**, **(h)** Dihedral FEL for residue 1 and 2 in water.

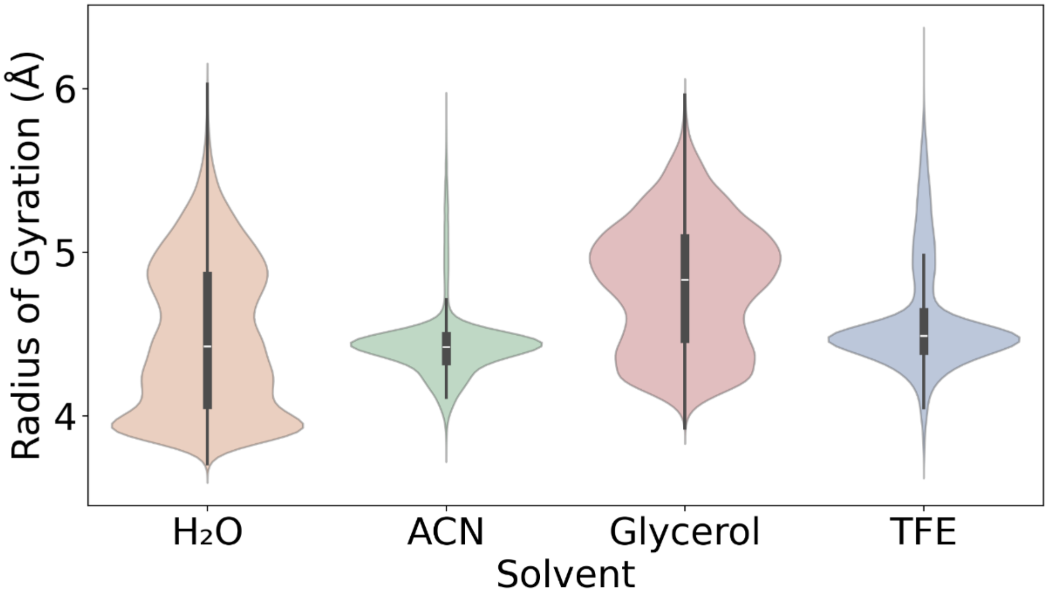


**Supplementary Figure 18**: Radius of gyration ($R_g$) distributions for MAAM in different solvents. TFE and acetonitrile favor more compact conformations relative to glycerol, with water showing intermediate behavior. These trends are consistent with backbone dihedral free-energy surfaces (Ramachandran plots).

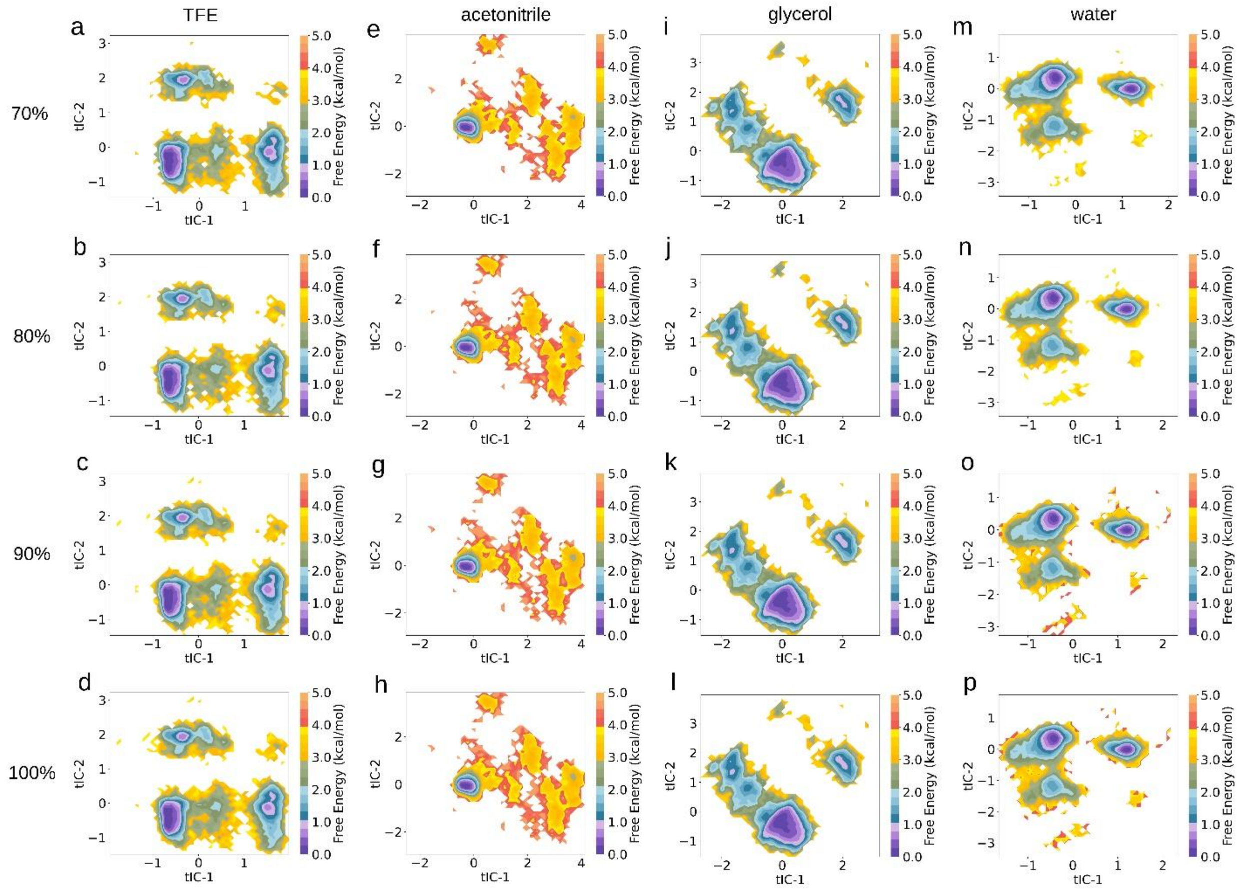


**Supplementary Figure 19**: TICA free energy landscapes (FELs) for MAAM constructed from randomly selected varying proportions of the sampled data. **(a)** TICA FEL for MAAM in TFE with 70% of sampled data **(b)** with 80% of sampled data **(c)** with 90% of sampled data **(d)** with the complete data. **(e)** TICA FEL for MAAM in acetonitrile with 70% of sampled data **(f)** with 80% of sampled data **(g)** with 90% of sampled data **(h)** with the complete data. **(i)** TICA FEL for MAAM in glycerol with 70% of sampled data **(j)** with 80% of sampled data **(k)** with 90% of sampled data **(l)** with the complete data. **(m)**TICA FEL for MAAM in water with 70% of sampled data **(n)** with 80% of sampled data **(o)** with 90% of sampled data **(p)** with the complete data.

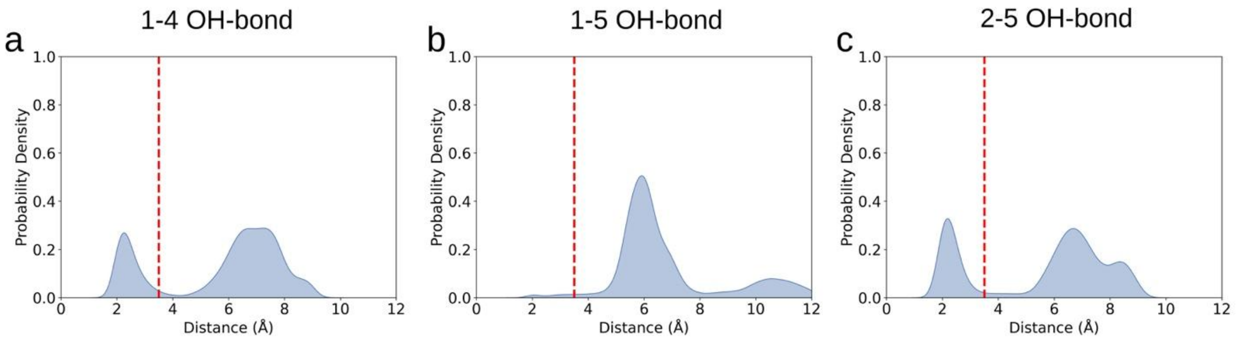


**Supplementary Figure 20**: Oxygen-Hydrogen bond distance distributions for MAAAM in TFE. **(a)** 1D kernel density estimation (KDE) plot for 1→4 OH distance **(b)** for 1→5 OH distance **(c)** for 2→5 OH distance for MAAAM in TFE**.** The red dashed line is for the distance value of 0.35nm, indicating the threshold for the hydrogen bond.

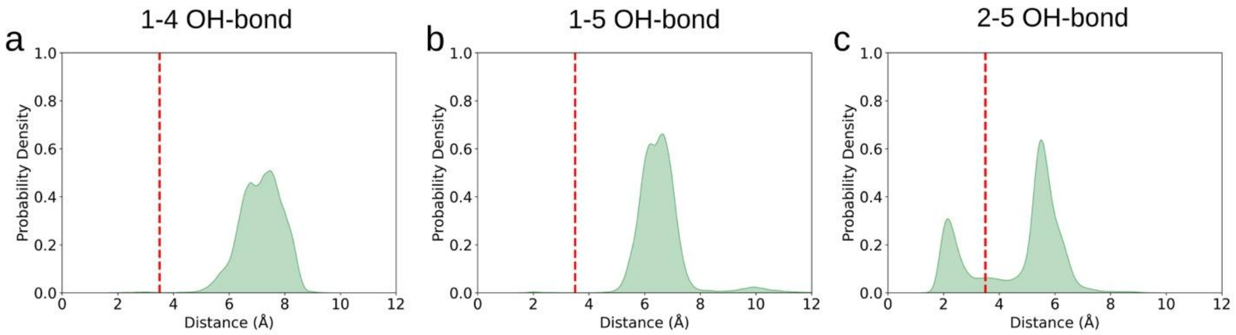


**Supplementary Figure 21**: Oxygen-Hydrogen bond distance distributions for MAAAM in acetonitrile. **(a)** 1D kernel density estimation (KDE) plot for 1→4 OH distance **(b)** for 1→5 OH distance **(c)** for 2→5 OH distance for MAAAM in acetonitrile**.** The red dashed line is for the distance value of 0.35nm, indicating the threshold for the hydrogen bond.

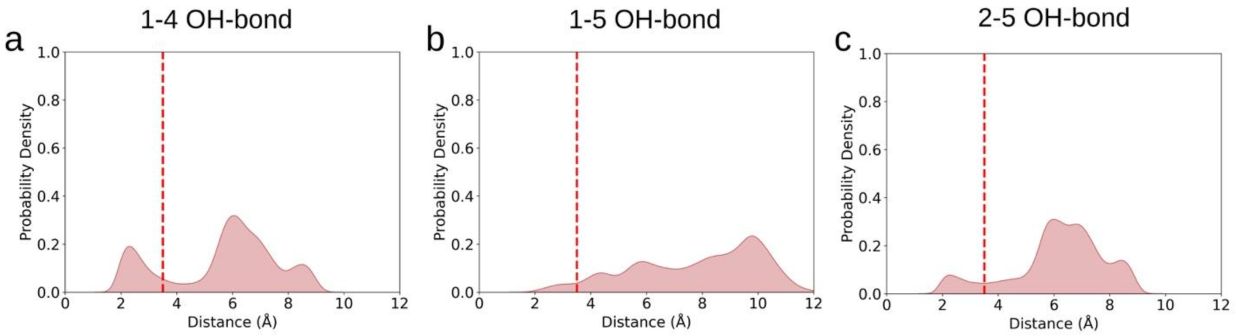


**Supplementary Figure 22**: Oxygen-hydrogen bond distance distributions for MAAAM in glycerol. **(a)** 1D kernel density estimation (KDE) plot for 1→4 OH distance **(b)** for 1→5 OH distance **(c)** for 2→5 OH distance for MAAAM in glycerol**.** The red dashed line is for the distance value of 0.35nm, indicating the threshold for the hydrogen bond.

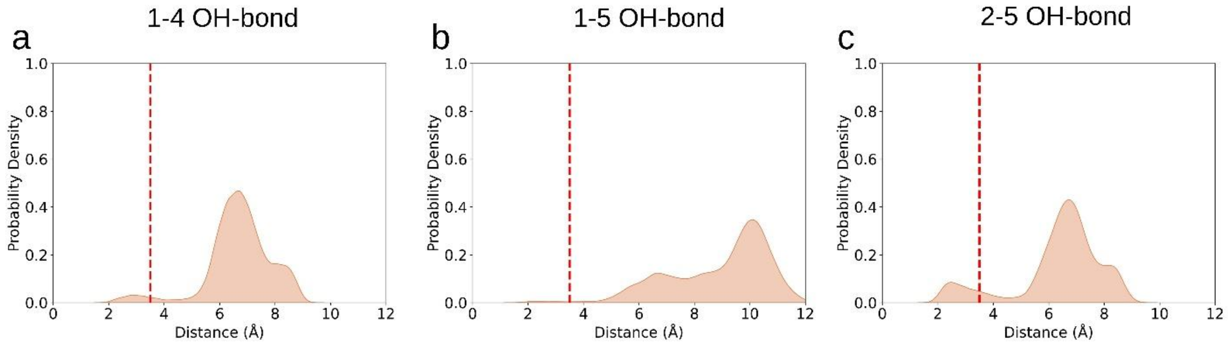


**Supplementary Figure 23**: Oxygen-hydrogen bond distance distributions for MAAAM in water. **(a)** 1D kernel density estimation (KDE) plot for 1→4 OH distance **(b)** for 1→5 OH distance **(c)** for 2→5 OH distance for MAAAM in water**.** The red dashed line is for the distance value of 0.35nm, indicating the threshold for the hydrogen bond. These results are in agreement, and reproduce prior published results[4]

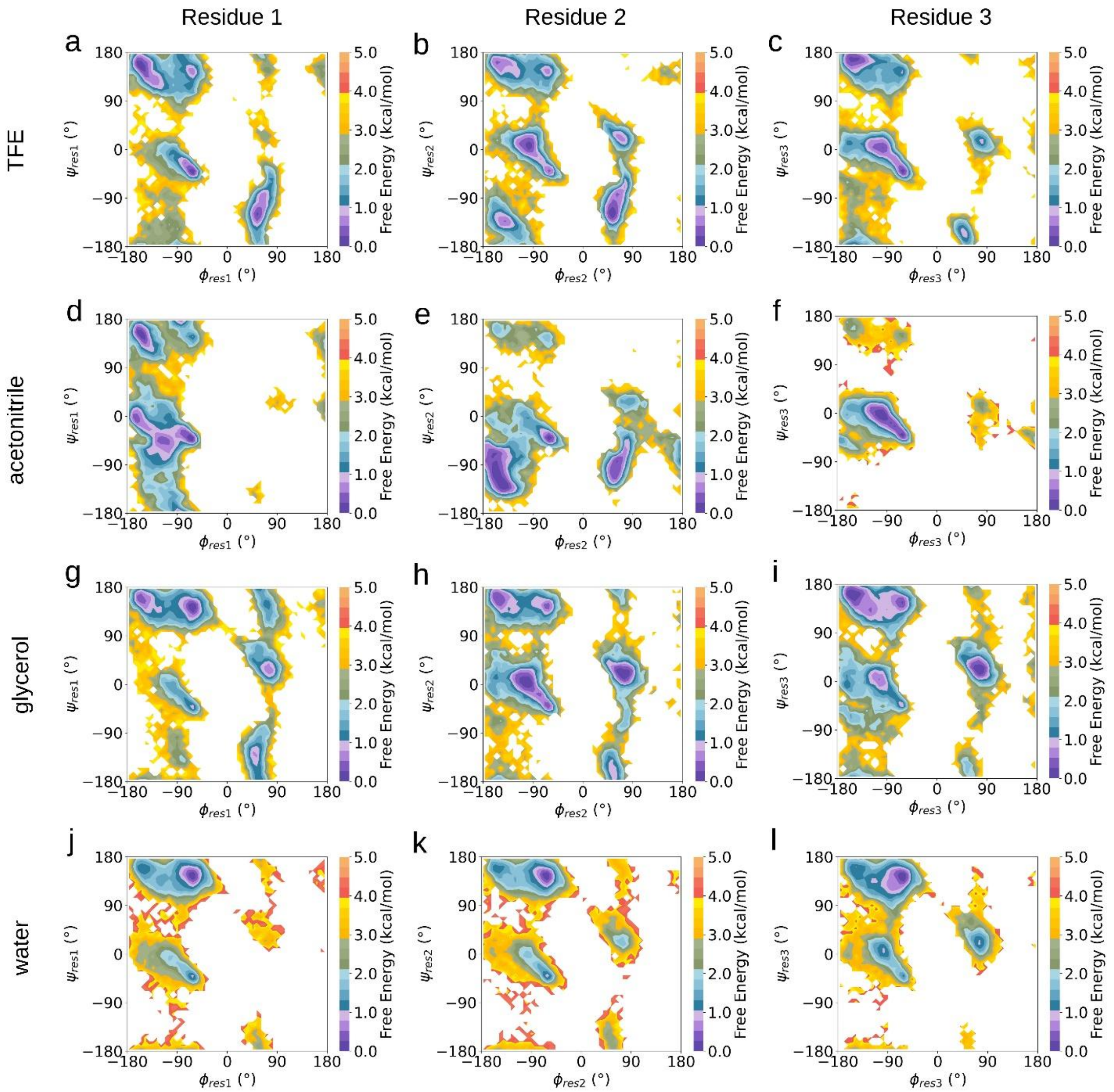


**Supplementary Figure 24**: Ramachandran dihedral free energy landscapes (FELs) for MAAAM pentapeptide in various solvents. Residues 1, 2 and 3 refer to the first, second and third central alanine residues respectively. **(a)** Dihedral FEL for Residue 1 in TFE **(b)** for Residue 2 in TFE **(c)** for Residue 3 in TFE. **(d)** Dihedral FEL for Residue 1 in acetonitrile **(e)** for Residue 2 in acetonitrile. **(f)** for Residue 3 in acetonitrile. **(g)** Dihedral FEL for Residue 1 in glycerol **(h)** for Residue 2 in glycerol **(i)** for Residue 3 in glycerol. **(j)** Dihedral FEL for Residue 1 in water **(k)** for Residue 2 in water **(l)** for Residue 3 in water.

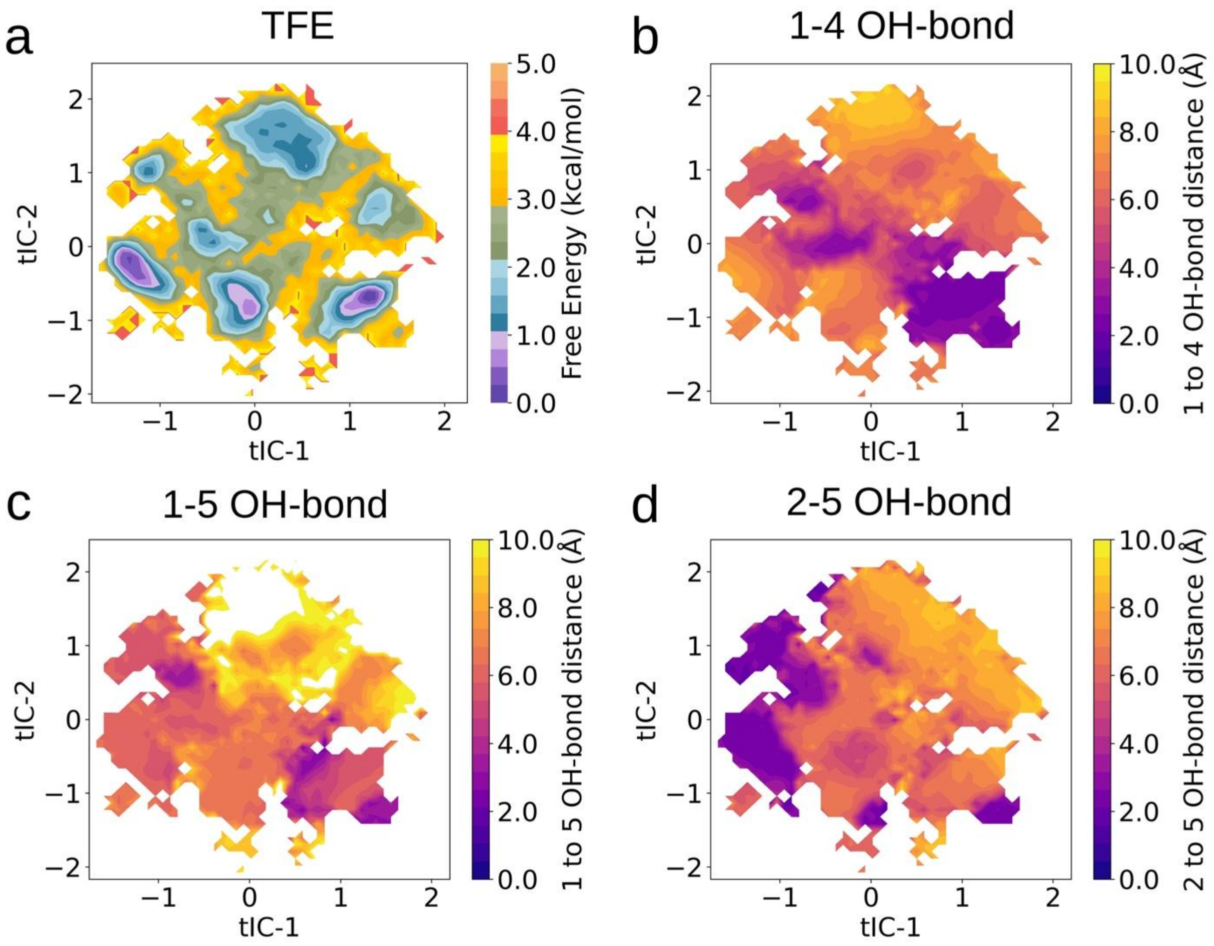


**Supplementary Figure 25**: TICA free energy landscape (FEL) for MAAAM in TFE. **(a)** TICA FEL for MAAAM in TFE projected on first two dimensions **(b)** TICA FEL for MAAAM in TFE mapped by 1→4 OH distance **(c)** mapped by 1→5 OH distance **(d)** mapped by 2→5 OH distance.

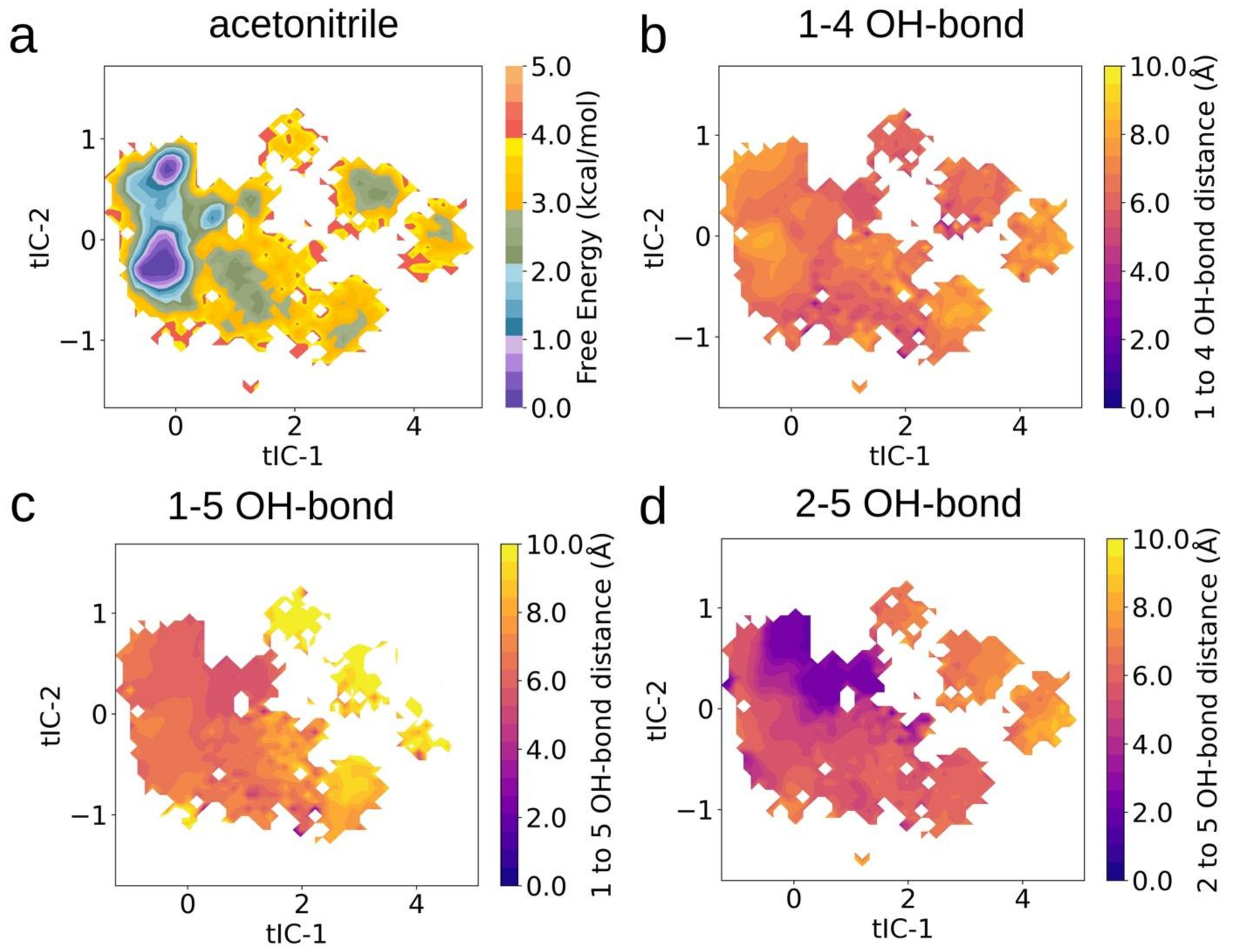


**Supplementary Figure 26**: TICA free energy landscape (FEL) for MAAAM in acetonitrile. **(a)** TICA FEL for MAAAM in acetonitrile projected on first two dimensions **(b)** TICA FEL for MAAAM in acetonitrile mapped by 1→4 OH distance **(c)** mapped by 1→5 OH distance **(d)** mapped by 2→5 OH distance.

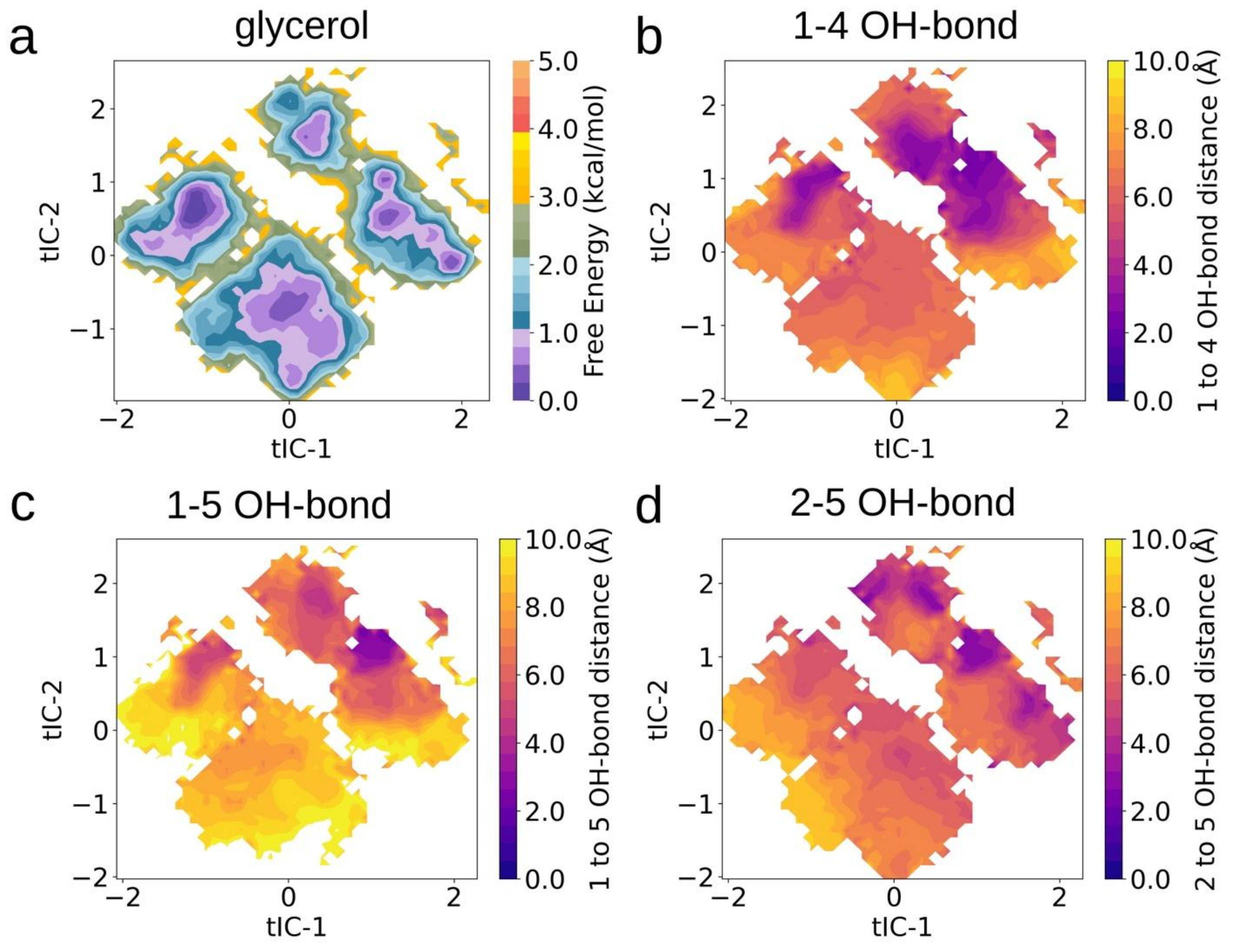


**Supplementary Figure 27**: TICA free energy landscape (FEL) for MAAAM in glycerol. **(a)** TICA FEL for MAAAM in glycerol projected on first two dimensions **(b)** TICA FEL for MAAAM in glycerol mapped by 1→4 OH distance **(c)** mapped by 1→5 OH distance **(d)** mapped by 2→5 OH distance.

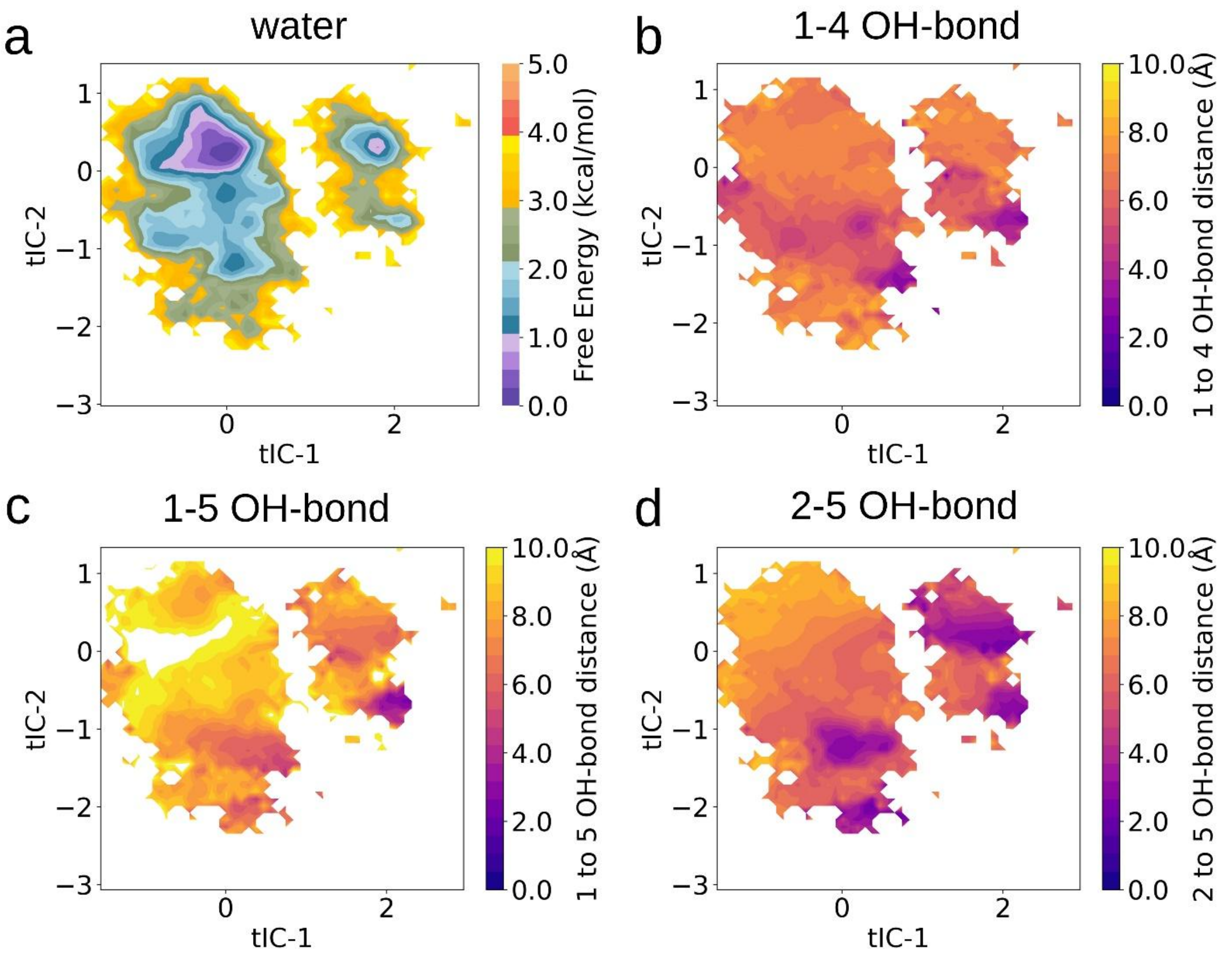


**Supplementary Figure 28**: TICA free energy landscape (FEL) for MAAAM in water. **(a)** TICA FEL for MAAAM in water projected on first two dimensions **(b)** TICA FEL for MAAAM in water mapped by 1→4 OH distance **(c)** mapped by 1→5 OH distance **(d)** mapped by 2→5 OH distance.

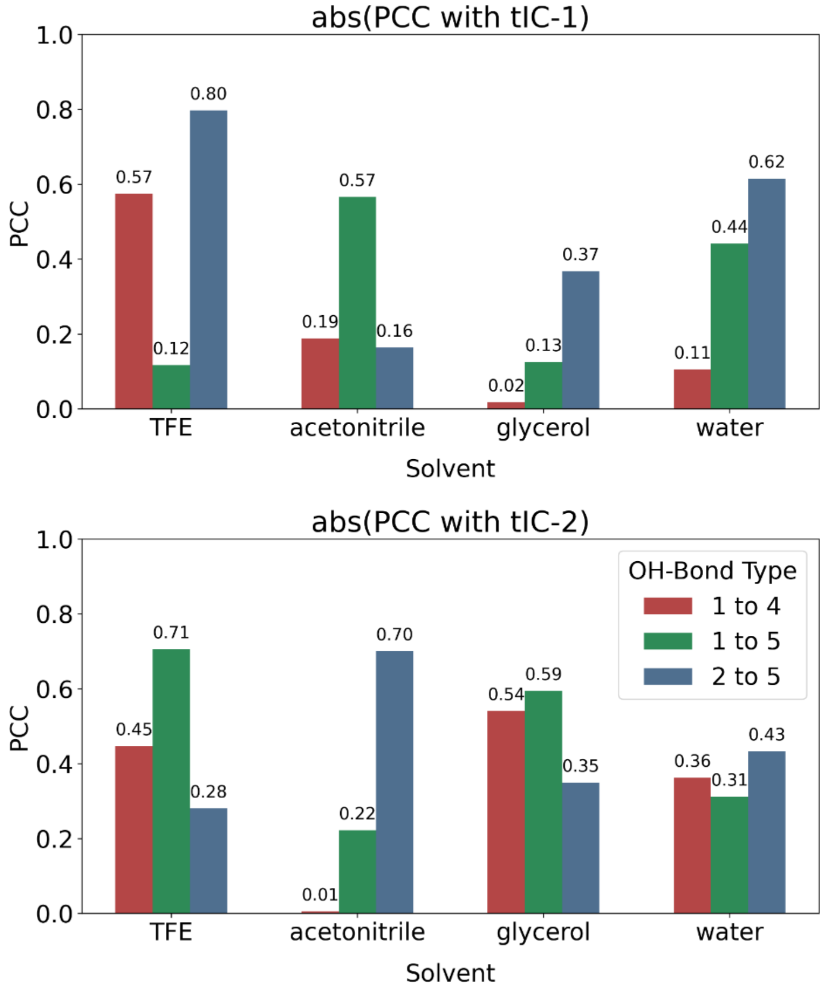


**Supplementary Figure 29**: Pearson correlation coefficients (PCCs) calculated between various OH-bond distances for MAAAM and tIC-1 and tIC-2. It should be noted that while tIC-1 (the slowest dynamical mode) is dominated by the 2→5 interaction for MAAAM in most solvents, the 1→4 hydrogen bond exhibits a significant correlation with tIC-2 (the second slowest mode), particularly in TFE (PCC = 0.45) and glycerol (PCC = 0.54).

## S8. Glycerol induced changes in conformational accessibility

To elucidate the molecular-scale electronic differences between MAAM and MAAAM in glycerol, we conducted further analysis of the MD simulations. This length-dependent effect likely arises from the unique conformational landscape imposed by glycerol. Unlike TFE or acetonitrile, which impose specific conformational preferences, glycerol creates a heterogeneous and kinetically sluggish ensemble in which peptides sample a wide range of geometries but interconvert slowly due to the solvent's permissive environment. Within this disordered landscape, the key distinction between MAAM and MAAAM lies in their intrinsic conformational sampling capabilities. MAAM, being shorter and more conformationally constrained, accesses a more limited set of geometries and therefore has fewer opportunities to adopt configurations that position the termini in favorable spatial proximity or orientations for efficient electrode coupling. In contrast, the additional alanine residue in MAAAM introduces an extra degree of freedom in the backbone, increasing the likelihood of transiently accessing geometries that support measurable electronic signatures. This effect emerges most clearly in glycerol because the solvent lacks strong conformational bias, allowing intrinsic backbone flexibility to dominate the observed behavior.

Taken together, these observations demonstrate that solvent effects on molecular-scale electronic signatures cannot be rationalized by dielectric constant alone. Although the dielectric constant (ε) captures bulk electrostatic screening, ET(30) values and H-bond donor and acceptor properties more directly describe the local solvation environment experienced by the peptide (**Supplementary Table 1**). Solvents such as TFE and acetonitrile impose strong and asymmetric H-bonding constraints that bias the conformational ensemble toward specific geometries. In contrast, glycerol provides a highly permissive H-bonding environment with minimal conformational bias. As a result, intrinsic backbone flexibility becomes the dominant factor governing conformational accessibility in glycerol, allowing differences between MAAM and MAAAM to emerge more clearly than in the other solvents examined.

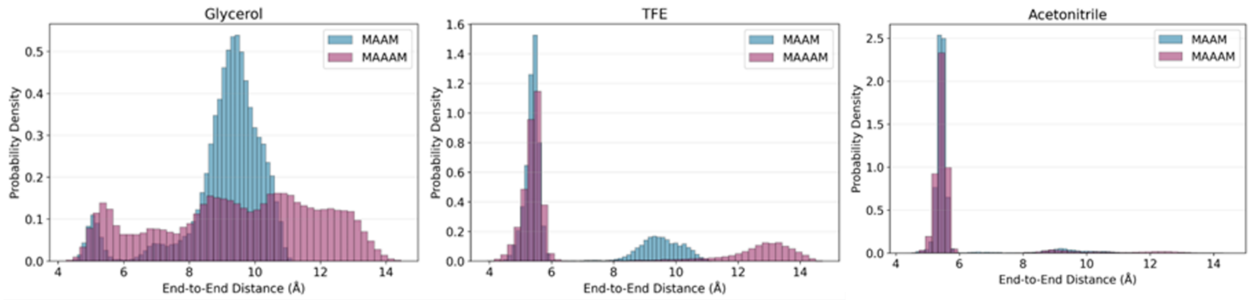


**Supplementary Figure 30**: End-to-end distance distributions for MAAM and MAAAM in various solvents. MAAAM samples a substantially broader conformational space in glycerol compared to the more focused distribution of MAAM. In contrast, other solvents impose tighter conformational constraints. Acetonitrile collapses both peptides to ~5 Å, while TFE stabilizes distinct bimodal populations.

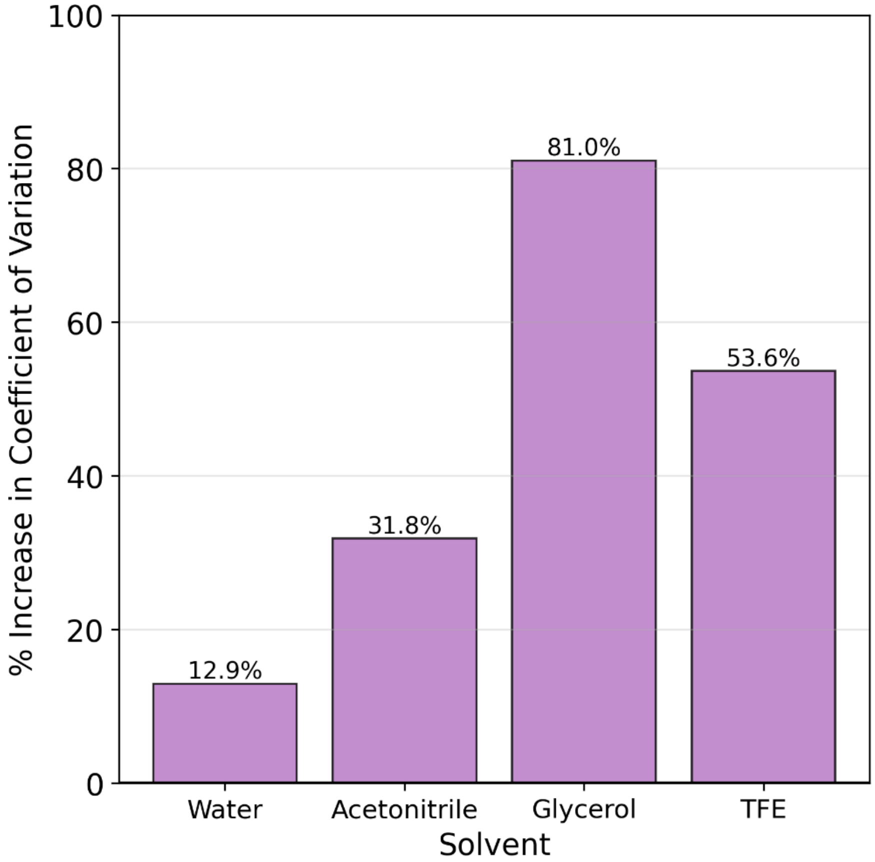


**Supplementary Figure 31**: Coefficient of variation of the end-to-end distance distributions in this solvent for MAAM versus MAAAM indicates that glycerol's environment allows the additional flexibility of MAAAM to manifest as occasional sampling of high-conductance geometries that the more rigid MAAM cannot readily access.

## S9. References


1. Li, S.; Yu, H.; Schwieter, K.; Chen, K.; Li, B.; Liu, Y.; Moore, J.S.; Schroeder, C.M. Charge transport and quantum interference effects in oxazole-terminated conjugated oligomers. *Journal of the American Chemical Society* **2019**, 141 (40), 16079-16084.
2. Li, B.; Yu, H.; Montoto, E.C.; Liu, Y.; Li, S.; Schwieter, K.; Rodríguez-López, J.; Moore, J.S.; Schroeder, C.M. Intrachain charge transport through conjugated donor–acceptor oligomers. *ACS Applied Electronic Materials* **2018**, 1 (1), 7-12.
3. Venkataraman, L.; Klare, J.E.; Tam, I.W.; Nuckolls, C.; Hybertsen, M.S.; Steigerwald, M.L. Single-molecule circuits with well-defined molecular conductance. *Nano Letters* **2006**, 6 (3), 458-462.
4. Samajdar, R.; Meigooni, M.; Yang, H.; Li, J.; Liu, X.; Jackson, N.E.; Mosquera, M.A.; Tajkhorshid, E.; Schroeder, C.M. Secondary structure determines electron transport in peptides. *Proceedings of the National Academy of Sciences* **2024**, 121 (32), e2403324121.
5. Liu, X.; Yang, H.; Harb, H.; Samajdar, R.; Woods, T.J.; Lin, O.; Chen, Q.; Romo, A.I.; Rodríguez-López, J.; Assary, R.S.; Moore, J.S.; Schroeder, C.M. Shape-persistent ladder molecules exhibit nanogap-independent conductance in single-molecule junctions. *Nature Chemistry* **2024**, 16 (11), 1772-1780.
6. Samajdar, R.; Liu, X.; Kuyama, K.; Kidokoro, Y.; Takeda, F.; Okamoto, I.; Kawahata, M.; Katagiri, K.; Moore, J.S.; Tanatani, A.; Schroeder, C.M. Aromatic amide foldamers show conformation-dependent electronic properties. *ChemPhysChem* **2025**, e202500672.
7. Nagahara, L.A.; Thundat, T.; Lindsay, S.M. Preparation and characterization of STM tips for electrochemical studies. *Review of Scientific Instruments* **1989**, 60, 3128-3130.
8. Stefani, D.; Guo, C.; Ornago, L.; Cabosart, D.; El Abbassi, M.; Sheves, M.; Cahen, D.; Van der Zant, H.S.J. Conformation-dependent charge transport through short peptides. *Nanoscale* **2021**, 13, 3002-3009.
9. Lin, L.; Tang, C.; Dong, G.; Chen, Z.; Pan, Z.; Liu, J.; Yang, Y.; Shi, J.; Ji, R.; Hong, W. Spectral clustering to analyze the hidden events in single-molecule break junctions. *The Journal of Physical Chemistry C* **2021**, 125 (6), 3623-3630.
10. Rousseeuw, P.J. Silhouettes: a graphical aid to the interpretation and validation of cluster analysis. *Journal of Computational and Applied Mathematics* **1987**, 20, 53-65.
11. David shaw, by E. et al. Anton, a Special-Purpose Machine for Molecular Dynamics Simulation. *Commun. of the ACM* **51**, 91-97 (2008).
12. Shirts, M. & Pande, V. S. Screen savers of the world unite. *Science* **290** 1903–1904 (2000).
13. Tien, M.Z.; Sydykova, D.K.; Meyer, A.G.; Wilke, C.O. PeptideBuilder: a simple Python library to generate model peptides. *PeerJ* **2013**, 1, e80.
14. Martínez, L.; Andrade, R.; Birgin, E.G.; Martínez, J.M. PACKMOL: a package for building initial configurations for molecular dynamics simulations. *Journal of Computational Chemistry* **2009**, 30, 2157-2164.
15. Jorgensen, W.L.; Chandrasekhar, J.; Madura, J.D.; Impey, R.W.; Klein, M.L. Comparison of simple potential functions for simulating liquid water. *The Journal of Chemical Physics* **1983**, 79 (2), 926-935.

16. Hatcher, E.R.; Guvench, O.; MacKerell, A.D. Jr. CHARMM additive all-atom force field for acyclic polyalcohols, acyclic carbohydrates, and inositol. *Journal of Chemical Theory and Computation* **2009**, 5, 1315-1327.
17. Vanommeslaeghe, K.; et al. CHARMM general force field: a force field for drug-like molecules compatible with the CHARMM all-atom additive biological force fields. *Journal of Computational Chemistry* **2010**, 31, 671-690.
18. Humphrey, W.; Dalke, A.; Schulten, K. VMD: visual molecular dynamics. *Journal of Molecular Graphics* **1996**, 14, 33-38.
19. Eastman, P.; et al. OpenMM 7: rapid development of high performance algorithms for molecular dynamics. *PLoS Computational Biology* **2017**, 13, e1005659.
20. Prasad, A.K.; Samajdar, R.; Panwar, A.S.; Martin, L.L. Origin of secondary structure transitions and peptide self-assembly propensity in trifluoroethanol–water mixtures. *The Journal of Physical Chemistry B* **2024**, 128 (32), 7736-7749.
21. Darden, T.; York, D.; Pedersen, L. Particle mesh Ewald: an N log (N) method for Ewald sums in large systems. *Journal of Chemical Physics* **1993**, 98, 10089-10089.
22. McGibbon, R.T.; et al. MDTraj: a modern open library for the analysis of molecular dynamics trajectories. *Biophysical Journal* **2015**, 109, 1528-1532.
23. Hoffmann, M.; et al. Deeptime: a Python library for machine learning dynamical models from time series data. *Machine Learning: Science and Technology* **2021**, 3, 015009.
24. Naritomi, Y.; Fuchigami, S. Slow dynamics in protein fluctuations revealed by time-structure based independent component analysis: the case of domain motions. *The Journal of Chemical Physics* **2011**, 134, 065101-065110.
25. Melo, M.C.R.; Bernardi, R.C.; De La Fuente-Nunez, C.; Luthey-Schulten, Z. Generalized correlation-based dynamical network analysis: a new high-performance approach for identifying allosteric communications in molecular dynamics trajectories. *The Journal of Chemical Physics* **2020**, 153 (13), 134.
26. Rodgers, J.L.; Nicewander, W.A. Thirteen ways to look at the correlation coefficient. *The American Statistician* **1988**, 42 (1), 59-66.
27. Uematsu, M.; Franck, E.U. Static dielectric constant of water and steam. *Journal of Physical and Chemical Reference Data* **1980**, 9 (4), 1291-1306.
28. Cerón-Carrasco, J.P.; Jacquemin, D.; Laurence, C.; Planchat, A.; Reichardt, C.; Sraïdi, K. Solvent polarity scales: determination of new ET(30) values for 84 organic solvents. *Journal of Physical Organic Chemistry* **2014**, *27*, 512–518.
29. Roccatano, D.; Colombo, G.; Fioroni, M.; Mark, A.E. Mechanism by which 2,2,2-trifluoroethanol/water mixtures stabilize secondary-structure formation in peptides: a molecular dynamics study. *Proceedings of the National Academy of Sciences* **2002**, 99 (19), 12179-12184.
30. Gagliardi, L.G.; Castells, C.B.; Rafols, C.; Rosés, M.; Bosch, E. Static dielectric constants of acetonitrile/water mixtures at different temperatures and Debye–Hückel A and $a_0B$ parameters for activity coefficients. *Journal of Chemical & Engineering Data* **2007**, 52 (3), 1103-1107.
31. Johari, G.P.; Whalley, E. Dielectric properties of glycerol in the range 0.1–$10^5$ Hz, 218–357 K, 0–53 kb. *Faraday Symposia of the Chemical Society* **1972**, 6, 23-41.
32. Abbott, A.P.; Harris, R.C.; Ryder, K.S.; D’Agostino, C.; Gladden, L.F.; Mantle, M.D. Glycerol eutectics as sustainable solvent systems. *Green Chemistry* **2011**, *13*, 82–90.
